# Path-Counting Formulas for Generalized Kinship Coefficients and Condensed Identity Coefficients

**DOI:** 10.1155/2014/898424

**Published:** 2014-07-21

**Authors:** En Cheng, Z. Meral Ozsoyoglu

**Affiliations:** ^1^Computer Science Department, The University of Akron, Akron, OH 44325, USA; ^2^Electrical Engineering and Computer Science Department, Case Western Reserve University, 10900 Euclid Avenue, Cleveland, OH 44106, USA

## Abstract

An important computation on pedigree data is the calculation of condensed identity coefficients, which provide a complete description of the degree of relatedness of two individuals. The applications of condensed identity coefficients range from genetic counseling to disease tracking. Condensed identity coefficients can be computed using linear combinations of generalized kinship coefficients for two, three, four individuals, and two pairs of individuals and there are recursive formulas for computing those generalized kinship coefficients (Karigl, 1981). Path-counting formulas have been proposed for the (generalized) kinship coefficients for two (three) individuals but there have been no path-counting formulas for the other generalized kinship coefficients. It has also been shown that the computation of the (generalized) kinship coefficients for two (three) individuals using path-counting formulas is efficient for large pedigrees, together with path encoding schemes tailored for pedigree graphs. In this paper, we propose a framework for deriving path-counting formulas for generalized kinship coefficients. Then, we present the path-counting formulas for all generalized kinship coefficients for which there are recursive formulas and which are sufficient for computing condensed identity coefficients. We also perform experiments to compare the efficiency of our method with the recursive method for computing condensed identity coefficients on large pedigrees.

## 1. Introduction

With the rapidly expanding field of medical genetics and genetic counseling, genealogy information is becoming increasingly abundant. In January 2009, the US Department of Health and Human Services released an updated and improved version of the Surgeon General's Web-based family health history tool [1]. This Web-based tool makes it easy for users to record their family health history. Large extended human pedigrees are very informative for linkage analysis. Pedigrees including thousands of members in 10–20 generations are available from genetically isolated populations [2, 3]. In human genetics, a pedigree is defined as “a simplified diagram of a family's genealogy that shows family members' relationships to each other and how a specific trait, abnormality, or disease has been inherited” [4]. Pedigrees are utilized to trace the inheritance of a specific disease, calculate genetic risk ratios, identify individuals at risk, and facilitate genetic counseling. To calculate genetic risk ratios or identify individuals at risk, we need to assess the degree of relatedness of two individuals. As a matter of fact, all measures of relatedness are based on the concept of* identical by descent* (IBD). Two alleles are identical by descent if one is an ancestral copy of the other or if they are both copies of the same ancestral allele. The IBD concept is primarily due to Cotterman [5] and Malecot [6] and has been successfully applied to many problems in population genetics.

The simplest measure of relationship between two individuals is their kinship coefficient. The* kinship coefficient* between two individuals *i* and *j* is the probability that an allele selected randomly from *i* and an allele selected randomly from the same autosomal locus of *j* are identical by descent. To better discriminate between different types of pairs of relatives, identity coefficients were introduced by Gillois [7] and Harris [8] and promulgated by Jacquard [9]. Considering the four alleles of two individuals at a fixed autosomal locus, there are 15 possible identity states. Disregarding the distinction between maternally and paternally derived alleles, we obtain 9 condensed identity states. The probabilities associated with each condensed identity state are called* condensed identity coefficients*, which are useful in a diverse range of fields. This includes the calculation of risk ratios for qualitative disease, the analysis of quantitative traits, and genetic counseling in medicine.

A recursive algorithm for calculating condensed identity coefficients proposed by Karigl [10] has been known for some time. This method requires that one calculates a set of generalized kinship coefficients, from which one obtains condensed identity coefficients via a linear transformation. One limitation is that this recursive approach is not scalable when applied to very large pedigrees. It has been previously shown that the kinship coefficients for two individuals [11–13] and the generalized kinship coefficients for three individuals [14, 15] can be efficiently calculated using path-counting formulas together with path encoding schemes tailored for pedigree graphs.

Motivated by the efficiency of path-counting formulas for computing the kinship coefficient for two individuals and the generalized kinship coefficient for three individuals, we first introduce a framework for developing path-counting formulas to compute generalized kinship coefficients concerning three individuals, four individuals, and two pairs of individuals. Then, we present path-counting formulas for all generalized kinship coefficients which have recursive formulas proposed by Karigl [10] and are sufficient to compute condensed identity coefficients. In summary, our ultimate goal is to use path-counting formulas for generalized kinship coefficients computation so that efficiency and scalability for condensed identity coefficients calculation can be improved.

The main contributions of our work are as follows:

- a framework to develop path-counting formulas for generalized kinship coefficients;
- a set of path-counting formulas for all generalized kinship coefficients having recursive formulas [10];
- experimental results demonstrating significant performance gains for calculating condensed identity coefficients based on our proposed path-counting formulas as compared to using recursive formulas [10].

## 2. Materials and Methods

This section describes kinship coefficients and generalized kinship coefficients, identity coefficients, and condensed identity coefficients in more detail. Conceptual terms for the path-counting formulas for three and four individuals are introduced in Section 2.3. In addition, an overview of path-counting formula derivation is presented.

### 2.1. Kinship Coefficients and Generalized Kinship Coefficients

The kinship coefficient between two individuals *a* and *b* is the probability that a randomly chosen allele at the same locus from each is identical by descent (IBD). There are two approaches to computing the kinship coefficient Φ_*ab*_: the recursive approach [10] and the path-counting approach [16]. The recursive formulas [10] for Φ_*ab*_ and Φ_*aa*_ are

(1) $$\begin{array}{c} \Phi_{ab}=\frac{1}{2}\left(\Phi_{fb}+\Phi_{mb}\right)\;\text{if}\;a\;\text{is}\;\text{not}\;\text{an}\;\text{ancestor}\;\text{of}\;b,\\ \Phi_{aa}=\frac{1}{2}\left(1+\Phi_{fm}\right)=\frac{1}{2}\left(1+F_{a}\right), \end{array}$$

where *f* and *m* denote the father and the mother of *a*, respectively, and *F*_*a*_ is the inbreeding coefficient of *a*.

Wright's path-counting formula [16] for Φ_*ab*_ is

(2) $$\begin{array}{c} \Phi_{ab}=\underset{A}{\sum }\underset{\langle P_{Aa},P_{Ab}\rangle \in PP}{\sum }{\left(\frac{1}{2}\right)}^{r+s+1}\left(1+F_{A}\right), \end{array}$$

where *A* is a common ancestor of *a* and *b*, *PP* is a set of nonoverlapping path-pairs 〈*P*_*Aa*_, *P*_*Ab*_〉 from *A* to *a* and *b*, *r* is the length of the path *P*_*Aa*_, *s* is the length of the path *P*_*Ab*_, and *F*_*A*_ is the inbreeding coefficient of *A*. The path-pair 〈*P*_*Aa*_, *P*_*Ab*_〉 is* nonoverlapping* if and only if the two paths share no common individuals, except *A*.

Recursive formulas proposed by Karigl [10] for generalized kinship coefficients concerning three individuals, four individuals, and two pairs of individuals are listed as follows in (3), (4), and (5):

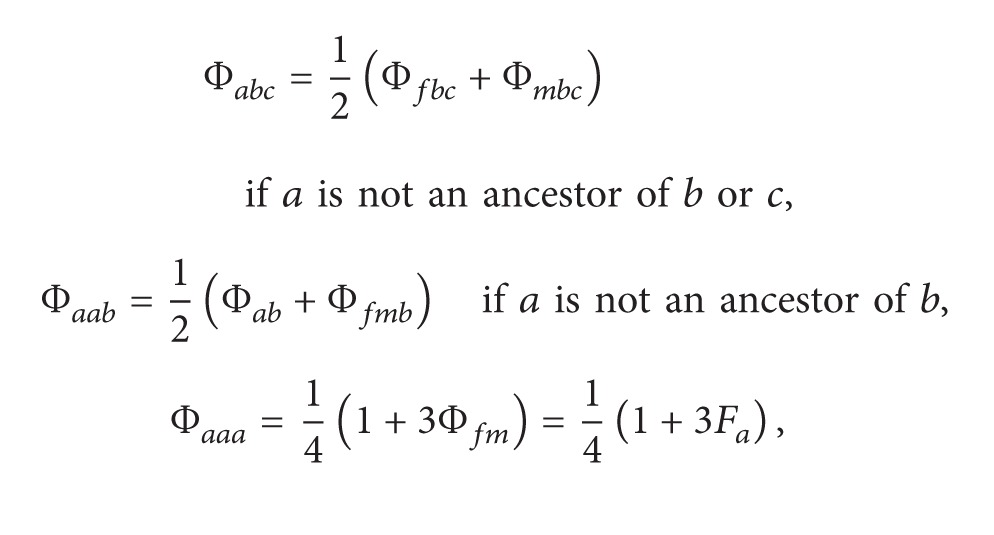
(3)

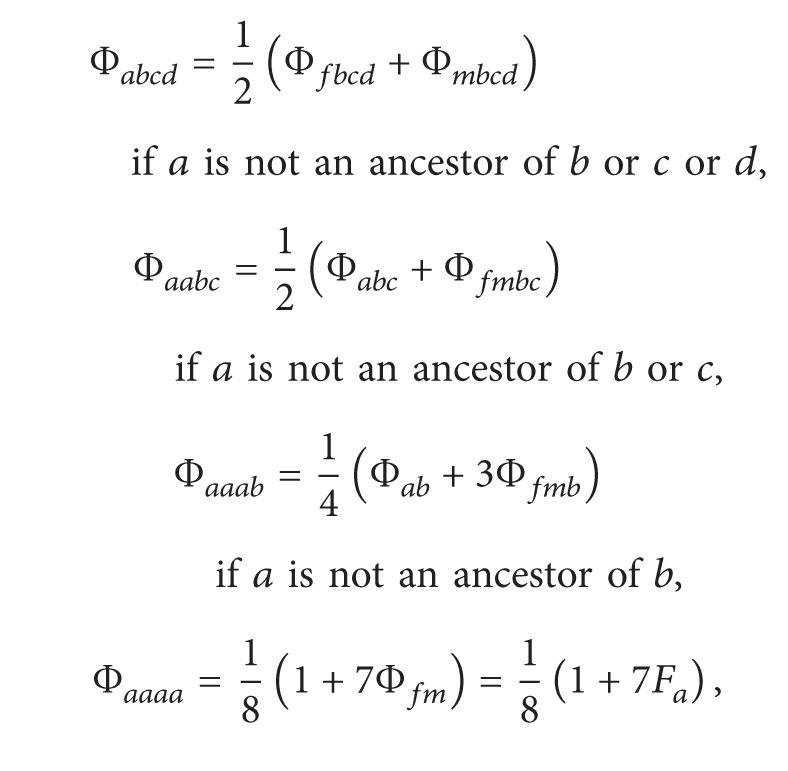
(4)

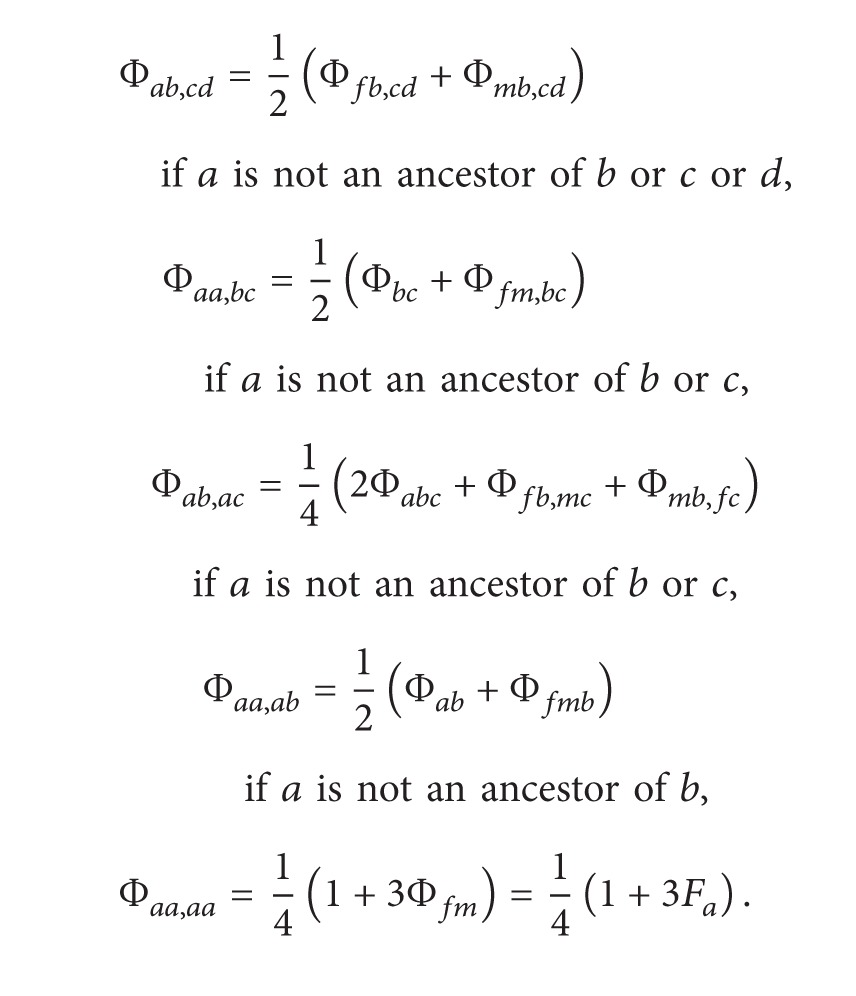
(5)


Φ_*abc*_ is the probability that randomly chosen alleles at the same locus from each of the three individuals (i.e., *a*, *b*, and *c*) are identical by descent (IBD). Similarly, Φ_*abcd*_ is the probability that randomly chosen alleles at the same locus from each of the four individuals (i.e., *a*, *b*, *c*, and *d*) are IBD. Φ_*ab*,*cd*_ is the probability that a random allele from *a* is IBD with a random allele from *b* and that a random allele from *c* is IBD with a random allele from *d* at the same locus. Note that Φ_*abc*_ = 0 if there is no common ancestor of *a*, *b*, and *c*. Φ_*abcd*_ = 0 if there is no common ancestor of *a*, *b*, *c*, and *d*, and Φ_*ab*,*cd*_ = 0 in the absence of a common ancestor either for *a* and *b* or for *c* and *d*.

### 2.2. Identity Coefficients and Condensed Identity Coefficients

**Figure 1:**
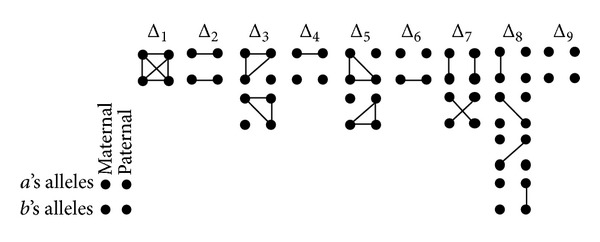
The 15 possible identity states for individuals *a* and *b*, grouped by their 9 condensed states. Lines indicate alleles that are IBD.

Given two individuals *a* and *b* with maternally and paternally derived alleles at a fixed autosomal locus, there are 15 possible identity states, and the probabilities associated with each identity state are called* identity coefficients*. Ignoring the distinction between maternally and paternally derived alleles, we categorize the 15 possible states to 9 condensed identity states, as shown in Figure 1. The states range from state 1, in which all four alleles are IBD, to state 9, in which none of the four alleles are IBD. The probabilities associated with each condensed identity state are called* condensed identity coefficients*, denoted by {Δ_*i*_∣1 ≤ *i* ≤ 9}  . The condensed identity coefficients can be computed based on generalized kinship coefficients using the linear transformation shown as follows in (6):

(6) $$\begin{array}{c} \left[\begin{array}{ccccccccc} 1 & 1 & 1 & 1 & 1 & 1 & 1 & 1 & 1\\ 2 & 2 & 2 & 2 & 1 & 1 & 1 & 1 & 1\\ 2 & 2 & 1 & 1 & 2 & 2 & 1 & 1 & 1\\ 4 & 0 & 2 & 0 & 2 & 0 & 2 & 1 & 0\\ 8 & 0 & 4 & 0 & 2 & 0 & 2 & 1 & 0\\ 8 & 0 & 2 & 0 & 4 & 0 & 2 & 1 & 0\\ 16 & 0 & 4 & 0 & 4 & 0 & 2 & 1 & 0\\ 4 & 4 & 2 & 2 & 2 & 2 & 1 & 1 & 1\\ 16 & 0 & 4 & 0 & 4 & 0 & 4 & 1 & 0 \end{array}\right]\left[\begin{array}{c} \Delta_{1}\\ \Delta_{2}\\ \Delta_{3}\\ \Delta_{4}\\ \Delta_{5}\\ \Delta_{6}\\ \Delta_{7}\\ \Delta_{8}\\ \Delta_{9} \end{array}\right]=\left[\begin{array}{c} 1\\ 2\Phi_{aa}\\ 2\Phi_{bb}\\ 4\Phi_{ab}\\ 8\Phi_{aab}\\ 8\Phi_{abb}\\ 16\Phi_{aabb}\\ 4\Phi_{aa,bb}\\ 16\Phi_{ab,ab} \end{array}\right]. \end{array}$$

In our work, we focus on deriving the path-counting formulas for the generalized kinship coefficients, including Φ_*abc*_, Φ_*abcd*_, and Φ_*ab*,*cd*_.

### 2.3. Terms Defined for Path-Counting Formulas for Three and Four Individuals

*(1) Triple-Common Ancestor.* Given three individuals *a*, *b*, and *c*, if *A* is a common ancestor of the three individuals, then we call *A* a* triple-common ancestor* of *a*, *b*, and *c*.

*(2) Quad-Common Ancestor*. Given four individuals *a*, *b*, *c*, and *d*, if *A* is a common ancestor of the four individuals, then we call *A* a* quad-common ancestor* of *a*, *b*, *c*, and *d*. 

*(3) P*(*A*, *a*). It denotes the set of all possible paths from *A* to *a*, where the paths can only traverse edges in the direction of parent to child such that *P*(*A*, *a*) ≠ *NULL* if and only if *A* is an ancestor of *a*. *P*_*Aa*_ denotes a particular path from *A* to *a*, where *P*_*Aa*_ ∈ *P*(*A*, *a*). 

*(4) Path-Pair.* It consists of two paths, denoted as 〈*P*_*Aa*_, *P*_*Ab*_〉, where *P*_*Aa*_ ∈ *P*(*A*, *a*) and *P*_*Ab*_ ∈ *P*(*A*, *b*). 

*(5) Nonoverlapping Path-Pair.* Given a path-pair 〈*P*_*Aa*_, *P*_*Ab*_〉, it is* nonoverlapping* if and only if the two paths share no common individuals, except *A*. 

*(6) Path-Triple.* It consists of three paths, denoted as 〈*P*_*Aa*_, *P*_*Ab*_, *P*_*Ac*_〉, where *P*_*Aa*_ ∈ *P*(*A*, *a*), *P*_*Ab*_ ∈ *P*(*A*, *b*), and *P*_*Ac*_ ∈ *P*(*A*, *c*). 

(*7) Path-Quad.* It consists of four paths, denoted as 〈*P*_*Aa*_, *P*_*Ab*_, *P*_*Ac*_, *P*_*Ad*_〉, where *P*_*Aa*_ ∈ *P*(*A*, *a*), *P*_*Ab*_ ∈ *P*(*A*, *b*), *P*_*Ac*_ ∈ *P*(*A*, *c*), and *P*_*Ad*_ ∈ *P*(*A*, *d*). 

(*8) Bi*_*C*(*P*_*Aa*_, *P*_*Ab*_). It denotes all common individuals shared between *P*_*Aa*_ and *P*_*Ab*_, except *A*.

(*9) Tri*_*C*(*P*_*Aa*_, *P*_*Ab*_, *P*_*Ac*_). It denotes all common individuals shared among *P*_*Aa*_, *P*_*Ab*_, and *P*_*Ac*_, except *A*.

(*10) Quad*_*C*(*P*_*Aa*_, *P*_*Ab*_, *P*_*Ac*_, *P*_*Ad*_). It denotes all common individuals shared among *P*_*Aa*_, *P*_*Ab*_, *P*_*Ac*_, and *P*_*Ad*_, except *A*.

(*11) Crossover and 2-Overlap Individual.* If *s* ∈ *Bi*_*C*(*P*_*Aa*_, *P*_*Ab*_), we call *s* a* crossover* individual with respect to *P*_*Aa*_ and *P*_*Ab*_ if the two paths pass through* different* parents of *s*. On the other hand, if *P*_*Aa*_ and *P*_*Ab*_ pass through the* same* parent of *s*, then we call *s* a 2-*overlap* individual with respect to *P*_*Aa*_ and *P*_*Ab*_. 

(*12) 3-Overlap Individual*. If *s* ∈ *Tri*_*C*(*P*_*Aa*_, *P*_*Ab*_, *P*_*Ac*_) and the three paths *P*_*Aa*_, *P*_*Ab*_, and *P*_*Ac*_ pass through the* same* parent of *s*, then we call *s* a* 3*-*overlap individual* with respect to *P*_*Aa*_, *P*_*Ab*_, and *P*_*Ac*_. 

*(13) 2-Overlap Path.* If *s* is a 2-overlap individual with respect to *P*_*Aa*_ and *P*_*Ab*_, then both *P*_*Aa*_ and *P*_*Ab*_ pass through the same parent of *s*, denoted by *p*, and the edge from *p* to *s* is called an* overlap* edge. All consecutive overlap edges constitute a path and this path is called a 2-*overlap path*. If the 2-overlap path extends all the way to the ancestor *A*, we call it a* root *2*-overlap path*. 

*(14) 3-Overlap Path.* It consists of all 3-overlap individuals in a consecutive order. If the 3-overlap path extends all the way to the root *A*, we call it a* root* 3*-overlap path*.

**Example 1 .**

**Figure 2:**
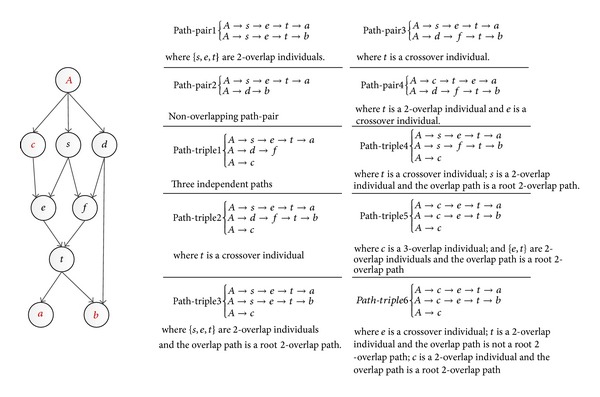
Examples of path-pairs and path-triples.

Consider the* path-pairs* from *A* to *a* and *b* in Figure 2, where *A* is a common ancestor of *a* and *b*. For* path-pair*1, *Bi*_*C*(*P*_*Aa*_, *P*_*Ab*_) = {*s*, *e*, *t*}, and *A*→*s*→*e*→*t* is a* root 2-overlap* path with respect to *P*_*Aa*_ and *P*_*Ab*_. For* path-pair*4, *Bi*_*C*(*P*_*Aa*_, *P*_*Ab*_) = {*e*, *t*}, where *e* is a* crossover* individual; *t* is a 2-*overlap individual* with respect to *P*_*Aa*_ and *P*_*Ab*_, and *e*→*t* is a* root 2-overlap* path with respect to *P*_*Aa*_ and *P*_*Ab*_.

**Example 2 .**

**Figure 3:**
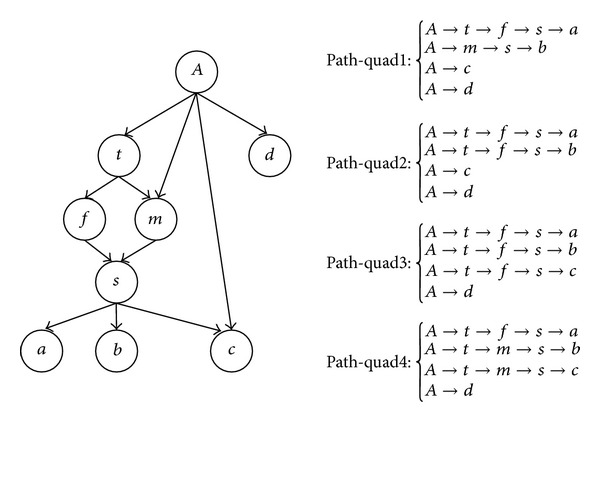
Examples of path-quads.

There are four path-quads listed in Figure 3, from *A* to four individuals *a*, *b*, *c*, and *d*, where *A* is a quad-common ancestor of the four individuals. For* path-quad2*, considering the paths *P*_*Aa*_ and *P*_*Ab*_, the path *A*→*t*→*f*→*s* is a* root *2*-overlap path*; {*t*, *f*, *s*} are 2*-overlap* individuals with respect to *P*_*Aa*_ and *P*_*Ab*_. For* path-quad3*, {*t*, *f*, *s*} are 3*-overlap* individuals with respect to *P*_*Aa*_, *P*_*Ab*_, and *P*_*Ac*_, and the path *A*→*t*→*f*→*s* is a* root 3-overlap path*.

Then, we summarize all the conceptual terms used in the path-counting formulas for two individuals, three individuals, and four individuals in Table 1 which reveals a glimpse of our framework for generalizing Wright's formula to three and four individuals from terminology aspect.

### 2.4. An Overview of Path-Counting Formula Derivation

According to Wright's path-counting formula [16] (see (2)) for two individuals *a* and *b*, the path-counting approach requires identifying common ancestors of *a* and *b* and calculating the contribution of each common ancestor to Φ_*ab*_. More specifically, for each common ancestor, denoted as *A*, we obtain all path-pairs from *A* to *a* and *b* and identify acceptable path-pairs. For Φ_*ab*_, an acceptable path-pair 〈*P*_*Aa*_, *P*_*Ab*_〉 is a nonoverlapping path-pair where the two paths share no common individuals, except *A*. In Figure 2,* path-pair2* is an acceptable path-pair, while* path-pair1*,* path-pair3*, and* path-pair4* are not acceptable path-pairs. The contribution of each common ancestor *A* to Φ_*ab*_ is computed based on the inbreeding coefficient of *A*, modified by the length of each acceptable path-pair.

To compute Φ_*abc*_, the path-counting approach requires identifying all triple-common ancestors of *a*, *b*, and *c* and summing up all triple-common ancestors' contributions to Φ_*abc*_. For each triple-common ancestor, denoted as *A*, we first identify all path-triples each of which consists of three paths from *A* to *a*, *b*, and *c*, respectively. Some examples of path-triples are presented in Figure 2.

For Φ_*ab*_, only nonoverlapping path-pairs are acceptable. A path-triple 〈*P*_*Aa*_, *P*_*Ab*_, *P*_*Ac*_〉 consists of three path-pairs 〈*P*_*Aa*_, *P*_*Ab*_〉, 〈*P*_*Aa*_, *P*_*Ac*_〉, and 〈*P*_*Ab*_, *P*_*Ac*_〉. For Φ_*abc*_, a path-triple might be acceptable even though either 2-overlap individuals or crossover individuals exist between a path-pair. The main challenge we need to address is finding necessary and sufficient conditions for acceptable path-triples.

Aiming at solving the problem of identifying acceptable path-triples, we first use a systematic method to generate all possible cases for a path-pair by considering different types of common individuals shared between the two paths. Then, we introduce building blocks which are connected graphs with conditions on every edge in the graph that encapsulates a set of acceptable cases of path-pairs. In each building block, we represent paths as nodes and interactions (i.e., shared common individuals between two paths) as edges. There are at least two paths in a building block. For each building block, we obtain all acceptable cases for concerned path-pairs. Given a path-triple, it can be decomposed to one or multiple building blocks. Considering a shared path-pair between two building blocks, we use the* natural join* operator from relational algebra to match the acceptable cases for the shared path-pair between two building blocks. In other words, considering the acceptable cases for building blocks as inputs, we use the natural join operator to construct all acceptable cases for a path-triple. Acceptable cases for a path-triple are identified and then used in deriving the path-counting formula for Φ_*abc*_.

**Figure 4:**
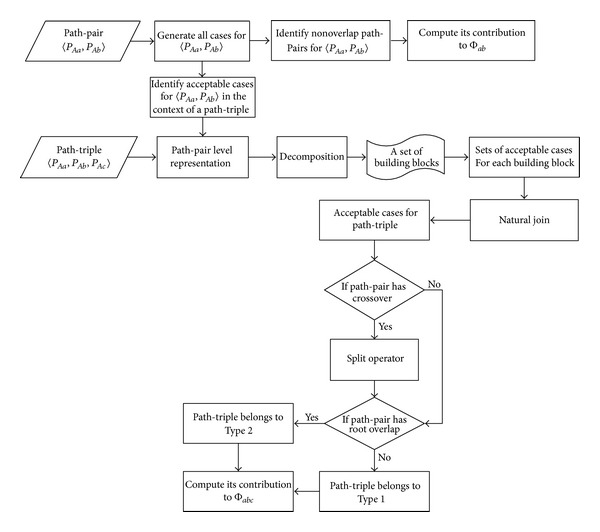
A flowchart for path-counting formula derivation.

Then, we summarize all the main procedures used for deriving the path-counting formula for Φ_*abc*_ in a flowchart shown in Figure 4. The main procedures are also applicable for deriving the path-counting formulas for Φ_*abcd*_ and Φ_*ab*,*cd*_.

## 3. Results and Discussion

### 3.1. Path-Counting Formulas for Three Individuals

We first introduce a systematic method to generate all possible cases for a path-pair. Then we discuss building blocks for path-triples and identify all acceptable cases which are used in deriving the path-counting formula for Φ_*abc*_.

#### 3.1.1. Cases for a Path-Pair

Given a path-pair 〈*P*_*Aa*_, *P*_*Ab*_〉 with *Bi*_*C*(*P*_*Aa*_, *P*_*Ab*_) ≠ *NULL*, where *A* is a common ancestor of *a* and *b* and *Bi*_*C*(*P*_*Aa*_, *P*_*Ab*_) consists of all common individuals shared between *P*_*Aa*_ and *P*_*Ab*_, except *A*, we introduce three patterns (i.e.,* crossover*, 2-*overlap,* and* root 2-overlap*) to generate all possible cases for 〈*P*_*Aa*_, *P*_*Ab*_〉.

1. *X*(*P*_*Aa*_, *P*_*Ab*_): *P*_*Aa*_ and *P*_*Ab*_ share one or multiple crossover individuals.
2. *T*(*P*_*Aa*_, *P*_*Ab*_): *P*_*Aa*_ and *P*_*Ab*_ are root 2-overlapping from *A*, and the root 2-overlap path can have one or multiple 2-overlap individuals.
3. *Y*(*P*_*Aa*_, *P*_*Ab*_): *P*_*Aa*_ and *P*_*Ab*_ are overlapping but not from *A*, and the 2-overlap path can have one or multiple 2-overlap individuals.

Based on the three patterns, *X*(*P*_*Aa*_, *P*_*Ab*_), *T*(*P*_*Aa*_, *P*_*Ab*_), and *Y*(*P*_*Aa*_, *P*_*Ab*_), we use regular expressions to generate all possible cases for the path-pair 〈*P*_*Aa*_, *P*_*Ab*_〉. For convenience, we drop 〈*P*_*Aa*_, *P*_*Ab*_〉 and use *X*, *T*, and *Y* instead of patterns *X*(*P*_*Aa*_, *P*_*Ab*_), *T*(*P*_*Aa*_, *P*_*Ab*_), and *Y*(*P*_*Aa*_, *P*_*Ab*_), whenever there is no confusion. When *Bi*_*C*(*P*_*Aa*_, *P*_*Ab*_) ≠ *NULL*, the eight cases shown in (7) cover all possible cases for 〈*P*_*Aa*_, *P*_*Ab*_〉. The completeness of eight cases shown in (7) for 〈*P*_*Aa*_, *P*_*Ab*_〉 can be proved by induction on the total number of *T*, *X*, and *Y* appearing in 〈*P*_*Aa*_, *P*_*Ab*_〉. Using the pedigree in Figure 2, Cases 1–3 and Case 6 are illustrated in (8), (9), (10), and (11):

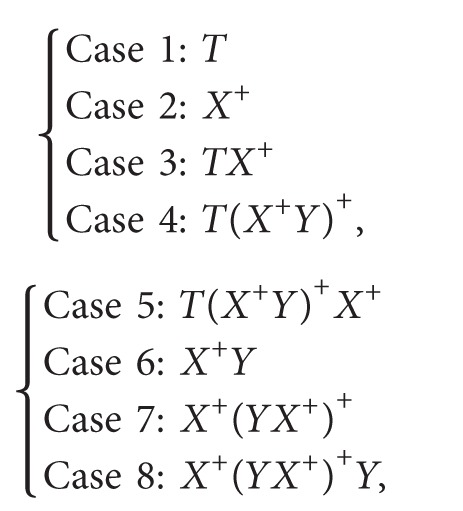
(7)

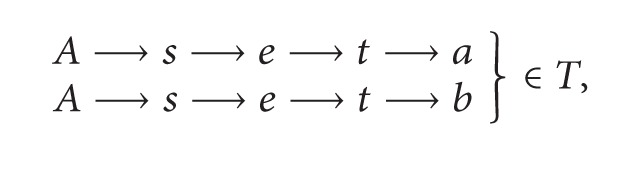
(8)
where {*s*, *e*, *t*} are 2-overlap individuals and the overlap path is a root 2-overlap path:

(9) A⟶s⟶e⟶t⟶aA⟶s⟶f⟶t⟶b}∈TX,

where *s* is a 2-overlap individual and the overlap path is a root 2-overlap path; *t* is a crossover individual:

(10) A⟶s⟶e⟶t⟶aA⟶d⟶f⟶t⟶b}∈X,

where *t* is a crossover individual:

(11) A⟶c⟶e⟶t⟶aA⟶s⟶e⟶t⟶b}∈XY,

where *e* is a crossover individual; *t* is a 2-overlap individual and the overlap path is a 2-overlap path.

#### 3.1.2. Path-Pair Level Graphical Representation of a Path-Triple

**Figure 5:**
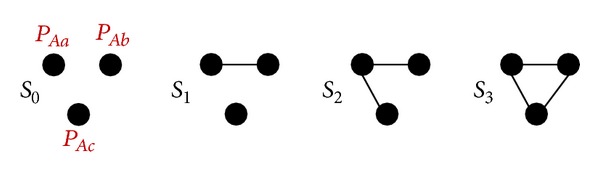
A path-pair level graphical representation of 〈*P*_*Aa*_, *P*_*Ab*_, *P*_*Ac*_〉.

Given a path-triple 〈*P*_*Aa*_, *P*_*Ab*_, *P*_*Ac*_〉, we represent each path as a node. The path-triple can be decomposed to three path-pairs (i.e., 〈*P*_*Aa*_, *P*_*Ab*_〉, 〈*P*_*Aa*_, *P*_*Ac*_〉, and 〈*P*_*Ab*_, *P*_*Ac*_〉). For each path-pair, if the two paths share at least one common individual (i.e., either 2-overlap individual or crossover individual), except *A*, then there is an edge between the two nodes representing the two paths. Therefore, we obtain four different scenarios *S*_0_–*S*_3_, shown in Figure 5.

In Figure 5, the scenario *S*_0_ has no edges, so it means that 〈*P*_*Aa*_, *P*_*Ab*_, *P*_*Ac*_〉 consists of three independent paths. In Figure 2,* path-triple*1 is an example of *S*_0_. Next, we introduce a lemma which can assist with identifying the options for the edges in the scenarios *S*_1_–*S*_3_.

**Lemma 3 .**

Given a path-triple 〈*P*_*Aa*_, *P*_*Ab*_, *P*_*Ac*_〉, consider the three path-pairs 〈*P*_*Aa*_, *P*_*Ab*_〉, 〈*P*_*Aa*_, *P*_*Ac*_〉, and 〈*P*_*Ab*_, *P*_*Ac*_〉, if there is a 2-overlap edge which is represented by *Y* in regular expression representation of any of the three path-pairs, and then the path-triple 〈*P*_*Aa*_, *P*_*Ab*_, *P*_*Ac*_〉 has no contribution to Φ_*abc*_.

**Proof**

In [17], Nadot and Vaysseix proposed, from a genetic and biological point of view, that Φ_*abc*_ can be evaluated by enumerating all eligible inheritance paths at allele-level starting from a triple common ancestor *A* to the three individuals *a*, *b*, and *c*.

**Figure 6:**
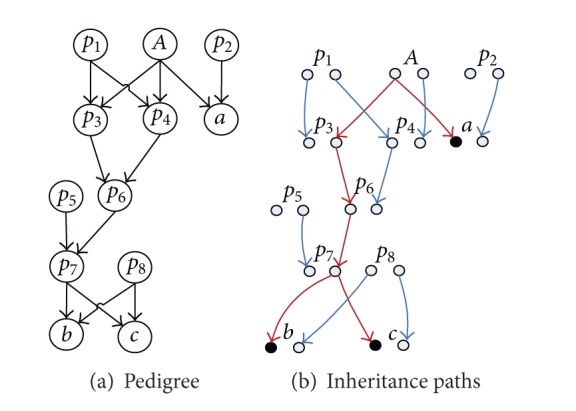
Examples of pedigree and inheritance paths.

For the pedigree in Figure 6, let us consider the path-triple 〈*P*_*Aa*_, *P*_*Ab*_, *P*_*Ac*_〉 listed as follows. *P*_*Aa*_ : *A* → *a*; *P*_*Ab*_ : *A* → *p*_3_ → *p*_6_ → *p*_7_ → *b*; *P*_*Ac*_ : *A* → *p*_4_ → *p*_6_ → *p*_7_ → *c*.

For 〈*P*_*Ab*_, *P*_*Ac*_〉,   *p*_6_ is a crossover individual, *p*_7_ is an overlap individual, and *p*_6_ → *p*_7_ is a 2-overlap edge represented by *Y* in regular expression representation (see the definition for *Y* in Section 3.1.1).

For the individual *p*_6_, let us denote the two alleles at one fixed autosomal locus as *g*_1_ and *g*_2_. At allele-level, only one allele can be passed down from *p*_6_ to *p*_7_. Since *p*_3_ and *p*_4_ are parents of *p*_6_, *g*_1_ is passed down from one parent, and *g*_2_ is passed down from the other parent. It is infeasible to pass down both *g*_1_ and *g*_2_ from *p*_6_ to *p*_7_. In other words, there are no corresponding inheritance paths for the path-triple 〈*P*_*Aa*_, *P*_*Ab*_, *P*_*Ac*_〉 with a 2-overlap edge between 〈*P*_*Ab*_, *P*_*Ac*_〉 (i.e., Case 6: *XY*). Therefore, such kind of path-triples has no contribution to Φ_*abc*_.

Figure 6(b) shows one example of eligible inheritance paths corresponding to a pedigree graph. Each individual is represented by two allele nodes. The eligible inheritance paths in Figure 6(b) consist of red edges only.

Only Case 1, Case 2, and Case 3 do not have *Y* in the regular expression representation of a path-pair (see (7)); considering the scenarios *S*_1_–*S*_3_ shown in Figure 5, an edge can have three options {Case  1:  *T*; Case  2:  *X*; Case  3:  *TX*}.

#### 3.1.3. Constructing Cases for a Path-Triple

**Figure 7:**
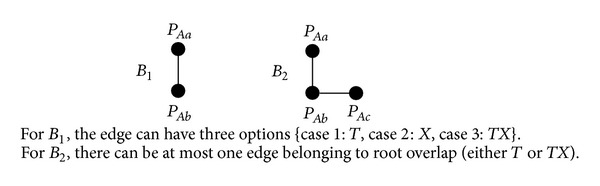
Building blocks {*B*_1_, *B*_2_} and basic rules.

For the scenarios *S*_1_–*S*_3_ in Figure 5, we define two building blocks {*B*_1_, *B*_2_} along with some rules in Figure 7 to generate acceptable cases. For *B*_1_, the edge can have three options {Case  1:  *T*; Case  2:  *X*; Case  3:  *TX*}. For *B*_2_, we cannot allow both edges to be root overlap, because if two edges are root overlap, then *P*_*Aa*_ and *P*_*Ac*_ must share at least one common individual, except *A*, which contradicts the fact that *P*_*Aa*_ and *P*_*Ac*_ have no edge.

**Figure 8:**
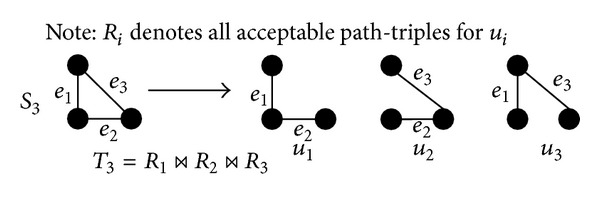
A graphical illustration for obtaining *T*_3_.

Next, we focus on generating all acceptable cases for the scenarios *S*_1_–*S*_3_ in Figure 5, where only *S*_3_ contains more than one building block. In order to leverage the dependency among building blocks, we decompose *S*_3_ to *S*_3_ = {*u*_1_ = *B*_2_, *u*_2_ = *B*_2_, *u*_3_ = *B*_2_}, shown in Figure 8. For each *u*_*i*_, we have a set of acceptable path-triples, denoted as *R*_*i*_.

Considering the dependency among {*R*_1_, *R*_2_, *R*_3_}, we use the natural join operator, denoted as ⋈, operating on {*R*_1_, *R*_2_, *R*_3_} to generate all acceptable cases for *S*_3_. As a result, we obtain *T*_3_ = *R*_1_⋈*R*_2_⋈*R*_3_, where *T*_3_ denotes the acceptable cases of the path-triple 〈*P*_*Aa*_, *P*_*Ab*_, *P*_*Ac*_〉 in the scenario *S*_3_.

For each scenario in Figure 5, we generate all acceptable cases for 〈*P*_*Aa*_, *P*_*Ab*_, *P*_*Ac*_〉. The scenario *S*_0_ has no edges, and it shows that 〈*P*_*Aa*_, *P*_*Ab*_, *P*_*Ac*_〉 consists of three independent paths, while, for the other scenarios *S*_*k*_ (*k* = 1,2, 3), the *k* edges can have two options:

1. all *k* edges belong to* crossover*; or
2. one edge belongs to* root 2-overlap*; the remaining (*k* − 1) edges belong to* crossover*.

In summary, acceptable path-triples can have at most one root 2-overlap path, any number of crossover individuals, but zero 2-overlap path.

#### 3.1.4. Splitting Operator

**Figure 9:**
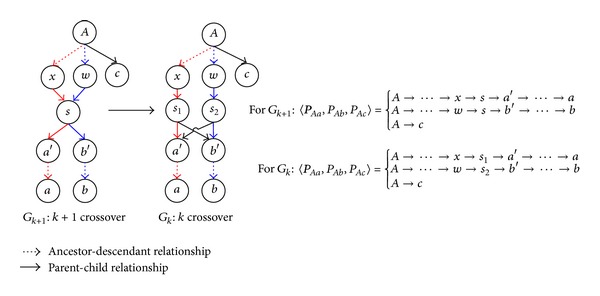
Transforming pedigree graph *G*_*k*_ + 1 having *k* + 1 crossover to *G*_*k*_ having *k* crossover.

Considering the existence of root 2-overlap path and crossover in acceptable path-triples, we propose a splitting operator to transform a path-triple with crossover individuals to a noncrossover path-triple without changing the contribution from this path-triple to Φ_*abc*_. The main purpose of using the splitting operator is to simplify the path-counting formula derivation process. We first use an example in Figure 9 to illustrate how the splitting operator works. In Figure 9, there is a crossover individual *s* between *P*_*Aa*_ and *P*_*Ab*_ in the path triple 〈*P*_*Aa*_, *P*_*Ab*_, *P*_*Ac*_〉 in *G*_*k*+1_. The splitting operator proceeds as follows:

1. split the node *s* to two nodes, *s*_1_ and *s*_2_;
2. transform the edges *s* → *a'* and *s* → *b'* to *s*_1_ → *a'* and *s*_2_ → *b'*, respectively;
3. add two new edges, *s*_2_ → *a'* and *s*_1_ → *b'*.

**Lemma 4 .**

Given a pedigree graph *G*_*k*+1_ having (*k* + 1) crossover individuals regarding 〈*P*_*Aa*_, *P*_*Ab*_, *P*_*Ac*_〉 shown in Figure 9, let *s* denote the lowest crossover individual, where no descendant of *s* can be a crossover individual among the three paths *P*_*Aa*_, *P*_*Ab*_, and *P*_*Ac*_. After using the splitting operator for the lowest crossover individual *s* in *G*_*k*_ + 1, the number of crossover individuals in *G*_*k*+1_ is decreased by 1.

**Proof**

The splitting operator only affects the edges from *s* to *a'* and *b'*. If there is a new crossover node appearing, the only possible node is either *a'* or *b'*. Assume *b'* becomes a crossover individual; it means that *b'* is able to reach *a* and *b* from two separate paths. It contradicts the fact that *s* is the lowest crossover individual between *P*_*Aa*_ and *P*_*Ab*_.

Next, we introduce a canonical graph which results from applying the splitting operator for all crossover individuals. The canonical graph has zero crossover individual.

**Definition 5 (Canonical Graph).**

Given a pedigree graph *G* having one or more crossover individuals regarding Φ_*abc*_, If there exists a graph *G'* which has no crossover individuals with regards to Φ_*abc*_ such that

- any acceptable path-triple in *G* has an acceptable path-triple in *G'* which has the same contribution to Φ_*abc*_ as the one in *G* for Φ_*abc*_;
- any acceptable path-triple in *G'* has an acceptable path-triple in *G* which and has the same contribution to Φ_*abc*_ as the one in *G'* for Φ_*abc*_.

We call *G'* a* canonical graph* of *G* regarding Φ_*abc*_.

**Lemma 6 .**

For a pedigree graph *G* having one or more crossover individuals regarding 〈*P*_*Aa*_, *P*_*Ab*_, *P*_*Ac*_〉, there exists a canonical graph *G'* for *G*.

**Proof**

The proof is by induction on the number of crossover individuals.

Induction hypothesis: assume that if *G* has *k* or less crossovers, there is a canonical graph *G'* for *G*.

In the induction step, let *G*_*k*+1_ be a graph with *k* + 1 crossovers; let *s* be the lowest crossover between paths *P*_*Aa*_ and *P*_*Ab*_ in *G*_*k*+1_. We apply the splitting operator on *s* in *G*_*k*+1_ and obtain *G*_*k*_ having *k* crossovers by Lemma 4.

#### 3.1.5. Path-Counting Formula for Φ_*abc*_

Now, we present the path-counting formula for Φ_*abc*_:

(12) Φabc=∑A(∑Type  1(12)LtripleΦAAA+∑Type  2(12)Ltriple+1ΦAA),

where  Φ_*AA*_ = (1/2)(1 + *F*_*A*_), Φ_*AAA*_ = (1/4)(1 + 3*F*_*A*_), *F*_*A*_: the inbreeding coefficient of *A*, *A*: a triple-common ancestor of *a*, *b*, and *c*,  Type 1: 〈*P*_*Aa*_, *P*_*Ab*_, *P*_*Ac*_〉 has zero root 2-overlap,  Type 2: 〈*P*_*Aa*_, *P*_*Ab*_, *P*_*Ac*_〉 has one root 2-overlap path *P*_*As*_ ending at the individual *s*

(13) Ltriple={LPAa+LPAb+LPAcfor    Type  1LPAa+LPAb+LPAc−LPAsfor    Type  2,

and *L*_*P*_*Aa*__: the length of the path *P*_*Aa*_ (also applicable for *P*_*Aa*_, *P*_*Ac*_, and *P*_*As*_).

For completeness, the path-counting formula for Φ_*aab*_ is given in Appendix A; and the correctness proof of the path-counting formula is given in Appendix B.

### 3.2. Path-Counting Formulas for Four Individuals

#### 3.2.1. Path-Pair Level Graphical Representation of 〈*P*_*Aa*_, *P*_*Ab*_, *P*_*Ac*_, *P*_*Ad*_〉

**Figure 10:**
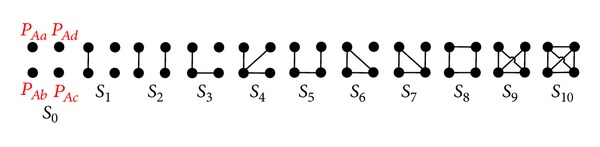
A path-pair level graphical representation of 〈*P*_*Aa*_, *P*_*Ab*_, *P*_*Ac*_, *P*_*Ad*_〉.

Given a path-quad 〈*P*_*Aa*_, *P*_*Ab*_, *P*_*Ac*_, *P*_*Ad*_〉 and *Quad*_*C*(*P*_*Aa*_, *P*_*Ab*_, *P*_*Ac*_, *P*_*Ad*_) = *∅*, the path-quad can have 11 scenarios *S*_0_–*S*_10_ shown in Figure 10 where all four paths are considered symmetrically.

**Figure 11:**
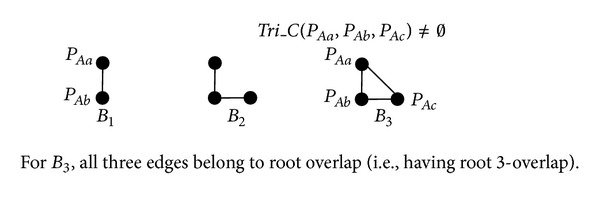
Building blocks for all scenarios of 〈*P*_*Aa*_, *P*_*Ab*_, *P*_*Ac*_, *P*_*Ad*_〉.

In Figure 11, we introduce three building blocks {*B*_1_, *B*_2_, *B*_3_}. For *B*_1_ and *B*_2_, the rules presented in Figure 7 are also applicable for Figure 11. For *B*_3_, we only consider root overlap, because the crossover individuals can be eliminated by using the splitting operator introduced in Section 3.1.4. Note that for *B*_3_, if *Tri*_*C*(*P*_*Aa*_, *P*_*Ab*_, *P*_*Ac*_) = *∅*, then it is equivalent to the scenario *S*_3_ in Figure 8 Therefore, we only need to consider *B*_3_ when *Tri*_*C*(*P*_*Aa*_, *P*_*Ab*_, *P*_*Ac*_) ≠ *∅*.

#### 3.2.2. Building Block-Based Cases Construction for 〈*P*_*Aa*_, *P*_*Ab*_, *P*_*Ac*_, *P*_*Ad*_〉

For a scenario *S*_*i*_  (0 ≤ *i* ≤ 10) in Figure 11, we first decompose *S*_*i*_ to one or multiple building blocks. For a scenario *S*_*i*_ ∈ {*S*_1_, *S*_3_}, it has only one building block, and all acceptable cases can be obtained directly. For *S*_2_ = {*u*_1_ = *B*_1_, *u*_2_ = *B*_1_}, there is no need to consider the conflict between the edges in *u*_1_ and *u*_2_ because *u*_1_ and *u*_2_ are disconnected. Let *R*_*i*_ denote all acceptable cases of the path-pairs in *u*_*i*_, and let *T*_*i*_ denote all acceptable cases for *S*_*i*_. Therefore, we obtain *T*_2_ = *R*_1_ × *R*_2_ where × denotes the Cartesian product operator from relational algebra.

For *S*_6_ = {*u*_1_ = *B*_3_}, we obtain *T*_6_ = *R*_1_. For *S*_*i*_ ∈ {*S*_*i*_∣4 ≤ *i* ≤ 10  and  *i* ≠ 6}, we define the largest subgraph of *S*_*i*_ based on which we construct *T*_*i*_.

**Definition 7 (Largest Subgraph).**

Given a scenario *S*_*i*_  (4 ≤ *i* ≤ 10 and *i* ≠ 6),* the largest subgraph* of *S*_*i*_, denoted as *S*_*j*_, is defined as follows:

1. *S*_*j*_ is a proper subgraph of *S*_*i*_;
2. if *S*_*i*_ contains *B*_3_, then *S*_*j*_ must also contain *B*_3_;
3. no such *S*_*k*_ exists that *S*_*j*_ is a proper subgraph of *S*_*k*_ while *S*_*k*_ is also a proper subgraph of *S*_*i*_.

For each scenario *S*_*i*_  (4 ≤ *i* ≤ 10 and *i* ≠ 6), we list the largest subgraph of *S*_*i*_, denoted as *S*_*j*_, in Table 2.

For a scenario *S*_*i*_  (4 ≤ *i* ≤ 10 and *i* ≠ 6), let Diff(*S*_*i*_∖*S*_*j*_) denote the set of building blocks in *S*_*i*_ but not in *S*_*j*_, where *S*_*j*_ is the largest subgraph of *S*_*i*_. Let |*E*_*i*_| and |*E*_*j*_| denote the number of edges in *S*_*i*_ and *S*_*j*_, respectively. According to Table 2, we can conclude that |*E*_*i*_ | −|*E*_*j*_ | = 1. In order to leverage the dependency among building blocks, we consider only *B*_2_ in Diff(*S*_*i*_∖*S*_*j*_). For example, Diff(*S*_5_∖*S*_3_) = {*B*_2_}. Let *T*_3_ denote all acceptable cases for *S*_3_. And let *R*_1_ denote the set of acceptable cases for Diff(*S*_5_∖*S*_3_). Then, we can use *S*_3_ and Diff(*S*_5_∖*S*_3_) to construct all acceptable cases for *S*_5_. Then, we apply this idea for constructing all acceptable cases for each *S*_*i*_ in Table 2.

Given a path-quad 〈*P*_*Aa*_, *P*_*Ab*_, *P*_*Ac*_, *P*_*Ad*_〉, an acceptable case has the following properties:

1. if there is one root 3-overlap path, there can be at most one root 2-overlap path;
2. otherwise, there can be at most two root 2-overlap paths.

#### 3.2.3. Path-Counting Formula for Φ_*abcd*_

Now, we present the path-counting formula for Φ_*abcd*_ as follows:

(14) Φabcd=∑A(∑Type  1(12)LquadΦAAAA+∑Type  2(12)Lquad+1ΦAAA+∑Type  3(12)Lquad+2ΦAA),

where   Φ_*AA*_ = (1/2)(1 + *F*_*A*_), Φ_*AAA*_ = (1/4)(1 + 3*F*_*A*_), Φ_*AAAA*_ = (1/8)(1 + 7*F*_*A*_), *F*_*A*_: the inbreeding coefficient of *A*, *A*: a quad-common ancestor of *a*, *b*, *c*, and *d*, Type 1: zero root 2-overlap and zero root 3-overlap path, Type 2: one root 2-overlap path *P*_*As*_ ending at *s*

(15) Type  3:{Case  1: two  root 2-overlap paths  PAs1, PAs2  ending  at  s1  and  s2,respectivelyCase  2: one  root 3-overlap path PAt  ending  at  tCase  3: one  root 2-overlap path PAs,one root 3-overlap  path  PAt  ending  at  s  and  t, respectively,Lquad={LPAa+LPAb+LPAc+LPAd  for    Type  1LPAa+LPAb+LPAc +LPAd−LPAsfor    Type  2LPAa+LPAb+LPAc+LPAd −LPAs1−LPAs2for  Case  1∈Type  3LPAa+LPAb+LPAc +LPAd−2∗LPAtfor  Case  2∈Type  3LPAa+LPAb+LPAc+LPAd −LPAt−LPAsfor  Case  3∈Type  3,

and *L*_*P*_*Aa*__: the length of the path *P*_*Aa*_ (also applicable for *P*_*Ab*_, *P*_*Ac*_, *P*_*Ad*_, etc.).

For completeness, the path-counting formulas for Φ_*aabc*_ and Φ_*aaab*_ are presented in Appendix A. The correctness of the path-counting formula for four individuals is proven in Appendix C.

### 3.3. Path-Counting Formulas for Two Pairs of Individuals

#### 3.3.1. Terminology and Definitions

*(1) 2-Pair-Path-Pair*. It consists of two pairs of path-pairs denoted as 〈(*P*_*Sa*_, *P*_*Sb*_), (*P*_*Tc*_, *P*_*Td*_)〉, where *P*_*Sa*_ ∈ *P*(*S*, *a*), *P*_*Sb*_ ∈ *P*(*S*, *b*), *P*_*Tc*_ ∈ *P*(*T*, *c*), *P*_*Td*_ ∈ *P*(*T*, *d*), *S* is a common ancestor of *a* and *b*, and *T* is a common ancestor of *c* and *d*. If *A* = *S* = *T*, then *A* is a* quad-common ancestor* of *a*, *b*, *c*, and *d*.

*(2) Homo-Overlap and Heter-Overlap Individual*. Given two pairs of individuals 〈*a*, *b*〉  and  〈*c*, *d*〉, if *s* ∈ *Bi*_*C*(*P*_*Aa*_, *P*_*Ab*_) (or *s* ∈ *Bi*_*C*(*P*_*Ac*_, *P*_*Ad*_), we call *s* a* homo-overlap individual* when *P*_*Aa*_ and *P*_*Ab*_ (or *P*_*Ac*_ and *P*_*Ad*_) pass through the* same* parent of *s*. If *r* ∈ *Bi*_*C*(*P*_*Ai*_, *P*_*Aj*_), where *i* ∈ {*a*, *b*} and *j* ∈ {*c*, *d*}, we call *r* a* heter-overlap individual* when *P*_*Ai*_ and *P*_*Aj*_ pass through the* same* parent of *r*. 

*(3) Root Homo-Overlap and Heter-Overlap Path.* Given a 2-pair-path-pair 〈(*P*_*Aa*_, *P*_*Ab*_), (*P*_*Ac*_, *P*_*Ad*_)〉, if *s* is a homo-overlap individual and the homo-overlap path extends all the way to the quad-common ancestor *A*, then we call it a* root homo-overlap path*. If *r* is a heter-overlap individual and the heter-overlap path extends all the way to the quad-common ancestor *A*, then we call it a* root heter-overlap path*.

**Example 8 .**

**Figure 12:**
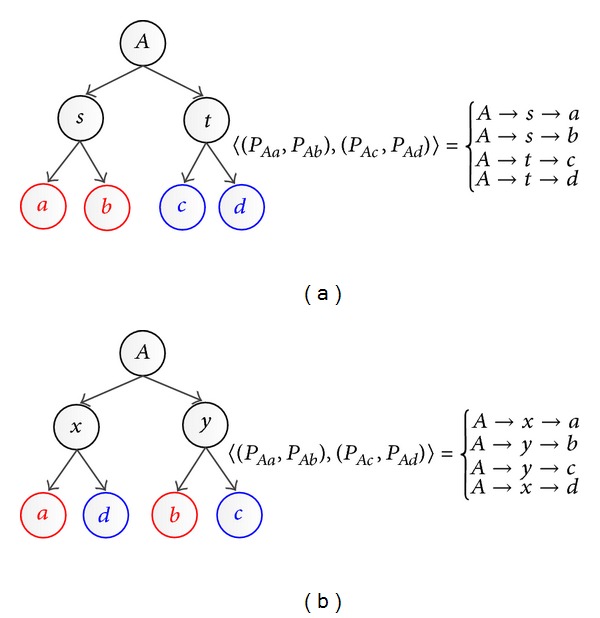
Examples of 2-pair-path-quads for Φ_*ab*,*cd*_.

*A* is* quad-common ancestor* for *a*, *b*, *c*, and *d* in Figure 12. For (a), *s* is a* homo-overlap* individual between *P*_*Aa*_ and *P*_*Ab*_.

*t* is a* homo-overlap* individual between *P*_*Ac*_ and *P*_*Ad*_. And, *A* → *s* and *A* → *t* are* root homo-overlap* paths. For (b), *x* is a* heter-overlap* individual between *P*_*Aa*_ and *P*_*Ad*_. *y* is a* heter-overlap* individual between *P*_*Ab*_ and *P*_*Ac*_. And *A* → *x* and *A* → *y* are* root heter-overlap* paths.

#### 3.3.2. Path-Counting Formula for Φ_*ab*,*cd*_

**Figure 13:**
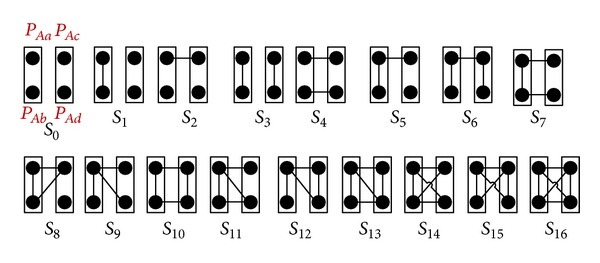
Scenarios of 〈(*P*_*Aa*_, *P*_*Ab*_), (*P*_*Ac*_, *P*_*Ad*_)〉 at path-pair level.

Now, we present a path-pair level graphical representation for 〈(*P*_*Aa*_, *P*_*Ab*_), (*P*_*Ac*_, *P*_*Ad*_)〉 shown in Figure 13. The options for an edge can be {*T*, *X*, *TX*}. (Refer to Section 3.1.1 for definitions of *T*, *X*, and *TX*). Based on the different types of 〈*P*_*Aa*_, *P*_*Ab*_, *P*_*Ac*_, *P*_*Ad*_〉 presented in (14), all cases for 〈(*P*_*Aa*_, *P*_*Ab*_), (*P*_*Ac*_, *P*_*Ad*_)〉 are summarized in Table 3, where *h* is the last individual of a root homo-overlap path *P*_*Ah*_ (i.e., the path *P*_*Ah*_ ending at *h*) and *r*_1_ and *r*_2_ are the last individuals of root heter-overlap paths *P*_*Ar*1_ and *P*_*Ar*2_, respectively.

Given a pedigree graph having one or multiple progenitors {*p*_*i*_∣*i* > 0}, we define that the generation of a progenitor *p*_*i*_ is 0, denoted as gen(*p*_*i*_) = 0. If an individual *a* has only one parent *p*, then we define gen(*a*) = gen(*p*) + 1. If an individual *a* has two parents *f* and *m*, we define gen(*a*) = MAX{gen(*f*), gen(*m*)} + 1.

The path-counting formula for Φ_*ab*,*cd*_ is as follows:

(16) Φab,cd=∑A(∑Type  1(12)L2-pairΦAAA+∑Type  2(12)L2-pair+1ΦAAA+∑Type  3(12)L2-pair+2ΦAA+∑Type  4(12)L2-pair+1ΦAA)+∑(S,T)∈Type  5(12)L〈PSa,PSb〉+L〈PTc,PTd〉+1ΦBB,

where *A*: a quad-common ancestor of *a*, *b*, *c*, and *d*, *S*: a common ancestor of *a* and *b*, and *T*: a common ancestor of *c* and *d*. For 〈(*P*_*Aa*_, *P*_*Ab*_), (*P*_*Ac*_, *P*_*Ad*_)〉  (*S* = *T* = *A*), there are four types (i.e., Type 1 to Type 4).

 Type 1: zero root homo-overlap and zero root heter-overlap.
 Type 2: zero root homo-overlap and one root heter-overlap *P*_*Ar*_ ending at *r*,
  (17) Type  3:{zero  root  homo-overlap  and  two  root   heter-overlap  PAr1  and PAr2  ending at r1   and  r2,respectively,one  root  homo-overlap  PAh  ending  at  h and  two  root  heter-overlap  PAr1  and  PAr2     ending  at  r1  and  r2,and  r1≠r2.
 Type 4: one root homo-overlap *P*_*Ah*_ ending at *h* and two root heter-overlap ending at *r*_1_ and  *r*_2_, and  *h* = *r*_1_ = *r*_2_. For 〈(*P*_*Sa*_, *P*_*Sb*_), (*P*_*Tc*_, *P*_*Td*_)〉  (*S* ≠ *T*), there is one type (i.e., Type 5).
 Type 5: 〈*P*_*Sa*_, *P*_*Sb*_〉 has zero overlap individual, 〈*P*_*Tc*_, *P*_*Td*_〉 has zero overlap individual.

At most one path-pair (either  〈*P*_*Sa*_, *P*_*Sb*_〉  or  〈*P*_*Tc*_, *P*_*Td*_〉)  can have crossover individuals.

Between a path from 〈*P*_*Sa*_, *P*_*Sb*_〉 and a path from 〈*P*_*Tc*_, *P*_*Td*_〉, there are no overlap individuals, but there can be crossover individuals, *x*, where *x* ≠ *S* and *x* ≠ *T*:

(18) B={Swhen  gen(S)<gen(T)Swhen  gen(S)=gen(T)   and  T  has  two  parentsTotherwise,L2-pair={LPAa+LPAb +LPAc+LPAdfor  Type  1LPAa+LPAb+LPAc +LPAd−LPArfor  Type  2LPAa+LPAb+LPAc +LPAd−LPAr1−LPAr2for  Type  3LPAa+LPAb+LPAc +LPAd−2∗LPAhfor  Type  4,L〈PSa,PSb〉=LPSa+LPSb for  Type    5,L〈PTc,PTd〉=LPTc+LPTd for  Type  5.

Note that if 〈*a*, *b*〉 and 〈*c*, *d*〉 have zero quad-common ancestors, we have the following formula for Φ_*ab*,*cd*_:

(19) $$\begin{array}{c} \Phi_{ab,cd}=\underset{\left(S,T\right)\in \text{Type}\;6}{\sum }{\left(\frac{1}{2}\right)}^{L_{\langle P_{Sa},P_{Sb}\rangle }+L_{\langle P_{Tc},P_{Td}\rangle }}\Phi_{SS}*\Phi_{TT}. \end{array}$$

Type  6:  〈*P*_*Sa*_, *P*_*Sb*_〉 is a nonoverlapping path-pair and 〈*P*_*Tc*_, *P*_*Td*_〉  is a nonoverlapping path-pair. Between a path from 〈*P*_*Sa*_, *P*_*Sb*_〉 and a path from 〈*P*_*Tc*_, *P*_*Td*_〉, there are no overlap individuals, but there can be crossover individuals.

*L*_〈*P*_*Sa*_,*P*_*Sb*_〉_ and *L*_〈*P*_*Tc*_,*P*_*Td*_〉_ are defined as in Type 5.

The correctness of the path-counting formula for Φ_*ab*.*cd*_ is proven in Appendix C. For completeness, please refer to [18] for the path-counting formulas for Φ_*aa*,*bc*_, Φ_*ab*,*ac*_, Φ_*ab*,*ab*_, and Φ_*aa*,*ab*_.

### 3.4. Experimental Results

In this section, we show the efficiency of our path-counting method using NodeCodes for condensed identity coefficients by making comparisons with the performance of a recursive method used in [10]. We implemented two methods: (1) using recursive formulas to compute each required kinship coefficient and generalized kinship coefficient; (2) using path-counting method coupled with NodeCodes to compute each required kinship coefficient and generalized kinship coefficient independently. We refer to the first method as* Recursive*, the second method as* NodeCodes*. For completeness, please refer to [18] for the details of the NodeCodes-based method.

Nodecodes of a node is a set of labels each representing a path to the node from its ancestors. Given a pedigree graph, let *r* be the progenitor (i.e., the node with 0 in-degree). (For simplicity, we assume there is one progenitor, *r*, as the ancestor of all individuals in the pedigree. Otherwise, a virtual node *r* can be added to the pedigree graph and all progenitors can be made children of *r*.)   For each node *u* in the graph, the set of NodeCodes of *u*, denoted as NC(*u*), are assigned using a breadth-first-search traversal starting from *r* as follows.

1. If *u* is *r* then NC(*r*) contains only one element: the empty string.
2. Otherwise, let *u* be a node with NC(*u*), and *v*_0_, *v*_1_, …, *v*_*k*_ be *u*'s children in sibling order; then for each *x* in NC(*u*), a code xi* is added to NC(*v*_*i*_), where 0 ≤ *i* ≤ *k*, and ∗ indicates the gender of the individual represented by node *v*_*i*_.

Computations of kinship coefficients for two individuals and generalized kinship coefficients for three individuals presented in [11, 12, 14, 15] are using NodeCodes. The NodeCodes-based computation schemes can also be applied for the generalized kinship coefficients for four individuals and two pairs of individuals. For completeness, please refer to [18] for the details using NodeCodes to compute the generalized kinship coefficients for four individuals and two pairs of individuals based on our proposed path-counting formulas in Sections 3.2 and 3.3.

In order to test the scalability of our approach for calculating condensed identity coefficients on large pedigrees, we used a population simulator implemented in [11] to generate arbitrarily large pedigrees. The population simulator is based on the algorithm for generating populations with overlapping generations in Chapter 4 of [19] along with the parameters given in Appendix B of [20] to model the relatively isolated Finnish Kainuu subpopulation and its growth during the years 1500–2000. An overview of the generation algorithm was presented in [11, 12, 14]. The parameters include starting/ending year, initial population size, initial age distribution, marriage probability, maximum age at pregnancy, expected number of children by time period, immigration rate, and probability of death by time period and age group.

We examine the performance of condensed identity coefficients using twelve synthetic pedigrees which range from 75 individuals to 195,197 individuals. The smallest pedigree spans 3 generations, and the largest pedigree spans 19 generations. We analyzed the effects of pedigree size and the depth of individuals in the pedigree (the longest path between the individual and a progenitor) on the computation efficiency improvement.

**Figure 14:**
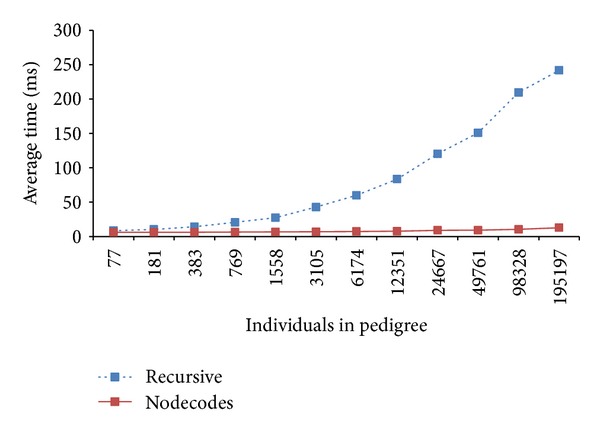
The effect of pedigree size on computation efficiency improvement.

In the first experiment, 300 random pairs were selected from each of our 12 synthetic pedigrees.  Figure 14 shows computation efficiency improvement for each pedigree. As can be seen, the improvement of* NodeCodes* over* Recursive* grew increasingly larger as the pedigree size increased, from a comparable amount of 26.83% on the smallest pedigree to 94.75% on the largest pedigree. It also shows that path-counting method coupled with NodeCodes can scale very well on large pedigrees in terms of computing condensed identity coefficients.

In our next experiment, we examined the effect of the depth of the individual in the pedigree on the query time. For each depth, we generated 300 random pairs from the largest synthetic pedigree.

**Figure 15:**
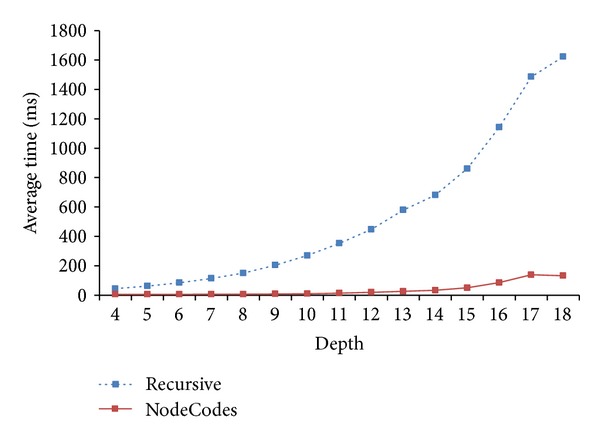
The effect of depth on computation efficiency improvement.

Figure 15 shows the effect of depth on the computation efficiency improvement. We can see the improvement of* NodeCodes* over* Recursive*, ranging from 86.48% to 91.30%.

## 4. Conclusion

We have introduced a framework for generalizing Wright's path-counting formula for more than two individuals. Aiming at efficiently computing condensed identity coefficients, we proposed path-counting formulas (PCF) for all generalized kinship coefficients for which are sufficient for expressing condensed identity coefficients by a linear combination. We also perform experiments to compare the efficiency of our method with the recursive method for computing condensed identity coefficients on large pedigrees. Our future work includes (i) further improvements on condensed identify coefficients computation by collectively calculating the set of generalized kinship coefficients to avoid redundant computations, and (ii) experimental results for using PCF in conjunction with encoding schemes (e.g., compact path-encoding schemes [13]) for computing condensed identity coefficients on very large pedigrees.

## Figures and Tables

**Figure 16:**
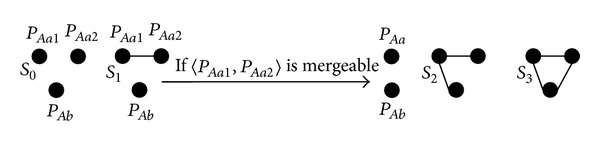
A path-pair level graphical representation of 〈*P*_*Aa*1_, *P*_*Aa*2_, *P*_*Ab*_〉.

**Figure 17:**
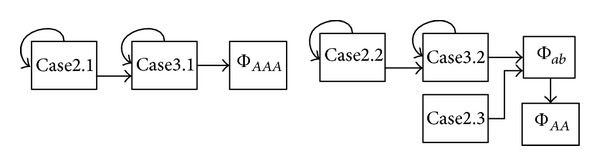
Dependency graph for different cases regarding Φ_*abc*_ and Φ_*aab*_.

**Figure 18:**
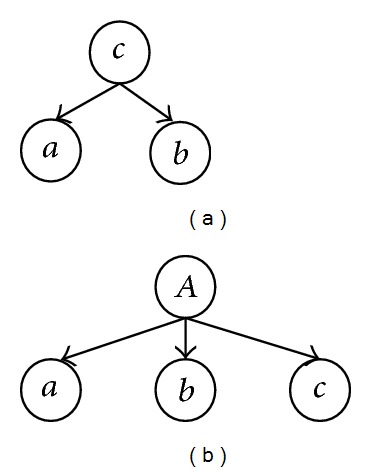
(a) *c* is a parent of *a* and *b*; (b) no individual is a parent of another.

**Figure 19:**
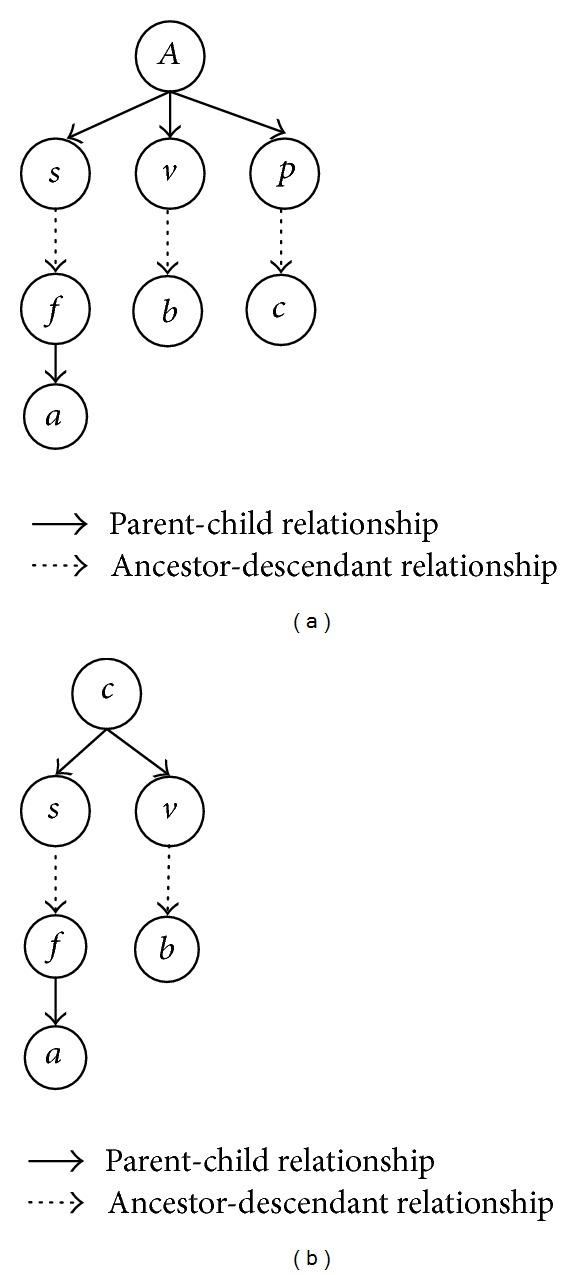
(a) No individual is a parent of another; (b) *c* is an ancestor of *a* and *b*.

**Figure 20:**
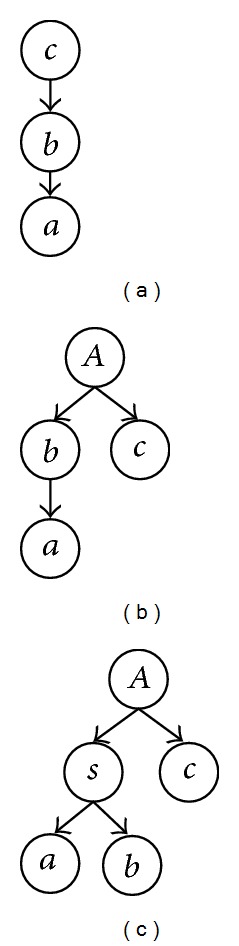
(a) *b* is a parent of *a*, and *c* is a parent of *b*; (b) *b* is a parent of *a*; (c) no individual who is a parent of another.

**Figure 21:**
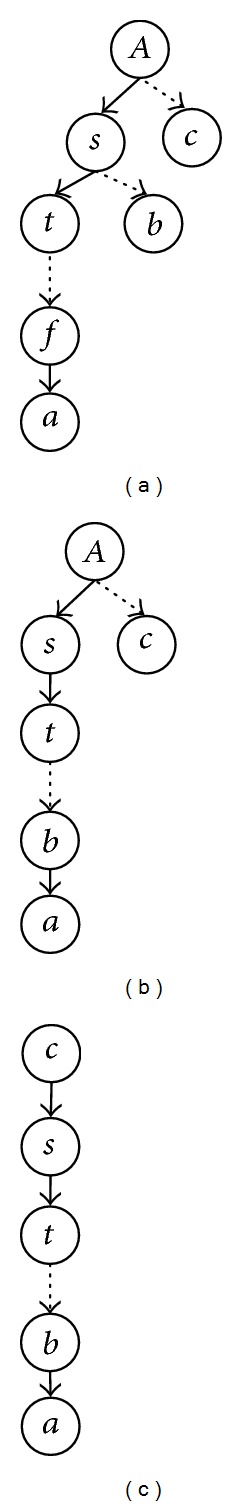
(a) No individual who is a parent of another; (b) *b* is a parent of *a*; (c) *b* is a parent of *a* and *c* is an ancestor of *b*.

**Figure 22:**
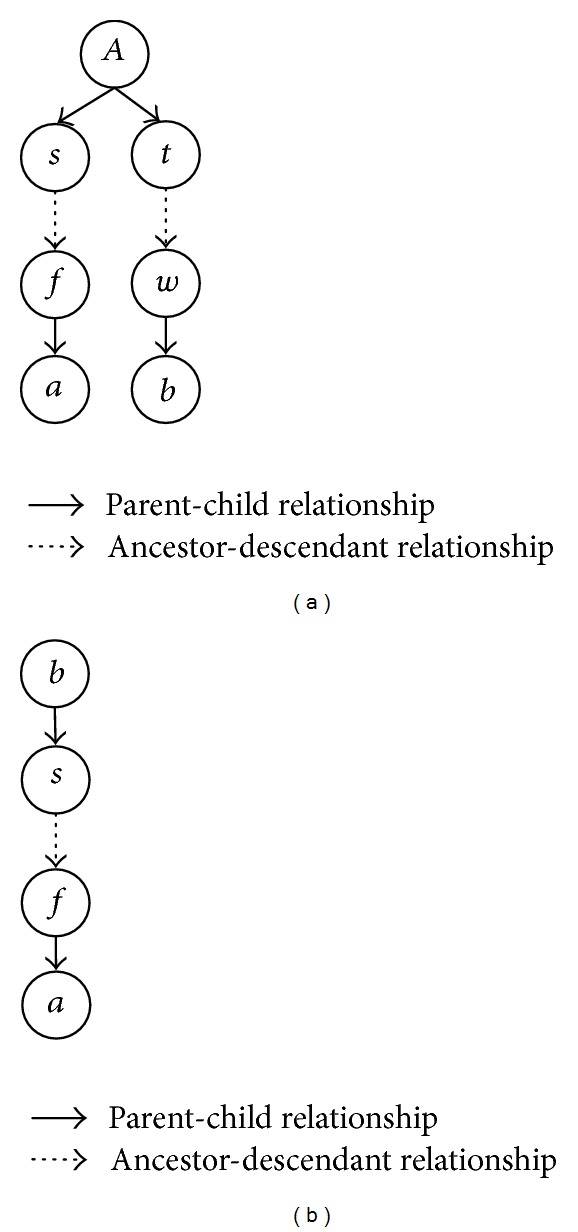
(a) *b* is not an ancestor of *a*; (b) *b* is an ancestor of *a*.

**Figure 23:**
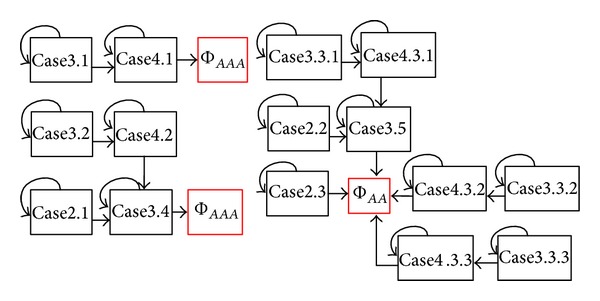
Dependency graph for different cases for four individuals.

**Figure 24:**
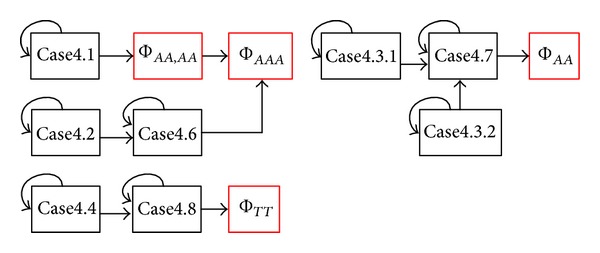
Dependency graph for different cases for two pairs of individuals.

**Table 1:** The conceptual terms used for two, three, and four individuals.

Two individuals	Three individuals	Four individuals
Common ancestor	Triple-common ancestor	Quad-common ancestor
Path-pair	Path-triple	Path-quad
*Bi*_*C*(*P*_*Aa*_, *P*_*Ab*_)	*Tr* *i*_*C*(*P*_*Aa*_, *P*_*Ab*_, *P*_*Ac*_)	*Qu* *ad*_*C*(*P*_*Aa*_, *P*_*Ab*_, *P*_*Ac*_, *P*_*Ad*_)
N/A	2-Overlap individual	3-Overlap individual
N/A	2-Overlap path	3-Overlap path
N/A	Root 2-overlap path	Root 3-overlap path
N/A	Crossover individual	Crossover individual

**Table 2:** Largest subgraph of a scenario *S*_*i*_ (4 ≤ *i* ≤ 10 and *i* ≠ 6).

*S*_*i*_	*S*_4_	*S*_5_	*S*_7_	*S*_8_	*S*_9_	*S*_10_

*S*_*j*_	*S*_3_	*S*_3_	*S*_6_	*S*_5_	*S*_7_	*S*_9_

**Table 3:** A summary of all cases for 〈(*P*_*Aa*_, *P*_*Ab*_), (*P*_*Ac*_, *P*_*Ad*_)〉.

〈*P*_*Aa*_, *P*_*Ab*_, *P*_*Ac*_, *P*_*Ad*_〉	〈(*P*_*Aa*_, *P*_*Ab*_), (*P*_*Ac*_, *P*_*Ad*_)〉
Zero root 2-overlap and zero root 3-overlap	Zero root homo-overlap and zero root heter-overlap

One root 2-overlap path	One root homo-overlap and zero root heter-overlap
	Zero root homo-overlap and one root heter-overlap

Two root 2-overlap paths	Two root homo-overlaps and zero root heter-overlap
	Zero root homo-overlap and two root heter-overlaps

One root 3-overlap path	One root homo-overlap and two root heter-overlaps, and *h* = *r*_1_ = *r*_2_

One root 2-overlap and one root 3-overlap	One root homo-overlap and two root heter-overlaps, and *r*_1_ = *r*_2_ ≠ *h*
	One root homo-overlap and two root heter-overlaps, and *h* = *r*_1_ ≠ *r*_2_

## Appendices

### A. Path-Counting Formulas of Special Cases

#### A.1. Path-Counting Formula for Φ_*a**ab*_

For 〈*P*_*Aa*1_, *P*_*Aa*2_〉, we introduce a special case, where *P*_*Aa*1_ and *P*_*Aa*2_ are* mergeable*.

Definition A.1 A.1 (Mergeable Path-Pair). A path-pair 〈*P*_*Aa*1_, *P*_*Aa*2_〉 is* mergeable* if and only if the two paths *P*_*Aa*1_ and *P*_*Aa*2_ are completely identical.

Next, we present a graphical representation of 〈*P*_*Aa*1_, *P*_*Aa*2_, *P*_*Ab*_〉 in Figure 16.

Lemma A.2 A.2. For *S*_2_ and *S*_3_ in Figure 16,  〈*P*_*Aa*1_, *P*_*Aa*2_〉 cannot be a mergeable path-pair.

Proof For *S*_2_ and *S*_3_, if 〈*P*_*Aa*1_, *P*_*Aa*2_〉 is mergeable, then any common individual *s* between *P*_*Aa*1_ and *P*_*Ab*_ is also a shared individual between *P*_*Aa*2_ and *P*_*Ab*_. It means *s* ∈ *Tri*_*C*(*P*_*Aa*1_, *P*_*Aa*2_, *P*_*Ab*_) which contradicts the fact that *Tri*_*C*(*P*_*Aa*1_, *P*_*Aa*2_, *P*_*Ab*_) = *∅*. Considering all three scenarios in Figure 16, only *S*_1_ can have a mergeable path-pair 〈*P*_*Aa*1_, *P*_*Aa*2_〉 by Lemma A.2. Now, we present our path-counting formula for Φ_*a**ab*_ where *a* is not an ancestor of *b*:

(A.1) Φaab=∑A(∑Type  1(12)Ltriple−1ΦAAA+∑Type  2(12)LtripleΦAA+∑Type  3(12)L〈PAa,PAb〉+1ΦAA),

where *A*: a common ancestor of *a* and *b*. When 〈*P*_*Aa*1_, *P*_*Aa*2_〉 is not mergeable,

Type 1: 〈*P*_*Aa*1_, *P*_*Aa*2_, *P*_*Ab*_〉 has no root 2-overlap.
Type 2: 〈*P*_*Aa*1_, *P*_*Aa*2_, *P*_*Ab*_〉 has one root 2-overlap path *P*_*As*_ ending at the individual *s*.

When 〈*P*_*Aa*1_, *P*_*Aa*2_〉 is mergeable, Type 3: 〈*P*_*Aa*_, *P*_*Ab*_〉 is a nonoverlapping path-pair

(A.2) Ltriple={LPAa1+LPAa2+LPAbfor  Type  1LPAa1+LPAa2+LPAb−LPAsfor  Type  2,L〈PAa,PAb〉=LPAa+LPAb for  Type  3.

For the sake of completeness, if *a* is an ancestor of *b*, there is no recursive formula for Φ_*a**ab*_ in [10], but we can use either the recursive formula for Φ_*ab**c*_ or the path-counting formula for Φ_*ab**c*_ to compute Φ_*a*1*a*2*b*_.

#### A.2. Path-Counting Formula for Φ_*a**ab**c*_

Given a path-quad 〈*P*_*Aa*1_, *P*_*Aa*2_, *P*_*Ab*_, *P*_*Ac*_〉, if 〈*P*_*Aa*1_, *P*_*Aa*2_〉 is not mergeable, then we process the path-quad as equivalent to 〈*P*_*Aa*_, *P*_*Ab*_, *P*_*Ac*_, *P*_*Ad*_〉. If 〈*P*_*Aa*1_, *P*_*Aa*2_〉 is mergeable, the path-quad 〈*P*_*Aa*1_, *P*_*Aa*2_, *P*_*Ab*_, *P*_*Ac*_〉 can be condensed to scenarios for 〈*P*_*Aa*_, *P*_*Ab*_, *P*_*Ac*_〉.

Now, we present a path-counting formula for Φ_*a**ab**c*_ where *a* is not an ancestor of *b* and *c* as follows:

(A.3) Φaabc=∑A(∑Type  1(12)Lquad−1ΦAAAA+∑Type  2(12)LquadΦAAA+∑Type  3(12)Lquad+1ΦAA)+∑A(∑Type  4(12)Ltriple+1ΦAAA+∑Type  5(12)Ltriple+2ΦAA),

where *A*: a quad-common ancestor of *a*, *b*, *c*, and *d*.

When 〈*P*_*Aa*1_, *P*_*Aa*2_〉 is not mergeable,

Type 1: zero root 2-overlap and zero root 3-overlap path;
Type 2: one root 2-overlap path *P*_*As*_ ending at *s*
  (A.4) Type 3:{Case  1: two root 2-overlap paths  PAs1 and PAs2  ending at s1  and  s2, respectivelyCase  2: one root 3-overlap path  PAt  ending at  tCase  3: one root 2-overlap  and one root 3-overlap paths PAs and  PAt  ending at s  and  t, respectively.

When 〈*P*_*Aa*1_, *P*_*Aa*2_〉 is mergeable,

Type 4: 〈*P*_*Aa*_, *P*_*Ab*_, *P*_*Ac*_〉 has zero root 2-overlap path;
Type 5: 〈*P*_*Aa*_, *P*_*Ab*_, *P*_*Ac*_〉 has one root 2-overlap path *P*_*As*_ ending at *s*
  (A.5) Lquad={LPAa1+LPAa2+LPAb+LPAcfor    Type  1LPAa1+LPAa2+LPAb+LPAc −LPAsfor    Type  2LPAa1+LPAa2+LPAb+LPAc −LPAs1−LPAs2for    Case  1∈Type  3LPAa1+LPAa2+LPAb+LPAc −LPAtfor  Case  2∈Type  3LPAa1+LPAa2+LPAb+LPAc −LPAt−LPAsfor  Case  3∈Type  3,Ltriple={LPAa+LPAb+LPAcfor    Type  4LPAa+LPAb+LPAc−LPAsfor    Type  5.

Note that if *a* is an ancestor of either *b* or *c*, or both of them, then the path-counting formula of Φ_*ab**cd*_ is applicable to compute Φ_*a*1*a*2*bc*_.

#### A.3. Path-Counting Formula for Φ_*aa**ab*_

A special case of 〈*P*_*Aa*_1__, *P*_*Aa*_2__, *P*_*Aa*_3__〉 for 〈*P*_*Aa*_1__, *P*_*Aa*_2__, *P*_*Aa*_3__, *P*_*Ab*_〉 is introduced when 〈*P*_*Aa*_1__, *P*_*Aa*_2__, *P*_*Aa*_3__〉 is mergeable. With the existence of a mergeable path-triple, 〈*P*_*Aa*_1__, *P*_*Aa*_2__, *P*_*Aa*_3__, *P*_*Ab*_〉 can be condensed to 〈*P*_*Aa*_, *P*_*Ab*_〉.

Definition A.3 A.3 (Mergeable Path-Triple). Given three paths *P*_*Aa*1_, *P*_*Aa*2_, and *P*_*Aa*3_, they are* mergeable* if and only if they are completely identical.

Lemma A.4 A.4. Given a path-quad 〈*P*_*Aa*_1__, *P*_*Aa*_2__, *P*_*Aa*_3__, *P*_*Ab*_〉, there must be at least* one mergeable path-pair* among 〈*P*_*Aa*_1__, *P*_*Aa*_2__〉, 〈*P*_*Aa*_1__, *P*_*Aa*_3__〉, 〈*P*_*Aa*_2__, *P*_*Aa*_3__〉.

Proof For an individual *a* with two parents *f* and *m*, the paternal allele of the individual *a* is transmitted from *f* and the maternal allele is transmitted from *m*. At allele level, only two descent paths starting from an ancestor are allowed. For a path-quad 〈*P*_*Aa*_1__, *P*_*Aa*_2__, *P*_*Aa*_3__, *P*_*Ab*_〉, there must be at least one mergeable path-pair among 〈*P*_*Aa*_1__, *P*_*Aa*_2__〉, 〈*P*_*Aa*_1__, *P*_*Aa*_3__〉, and 〈*P*_*Aa*_2__, *P*_*Aa*_3__〉.

For simplicity, we treat 〈*P*_*Aa*1_, *P*_*Aa*2_〉 as a default mergeable path-pair.

Now, we present the path-counting formula for Φ_*aa**ab*_ where *a* is not an ancestor of *b* as follows:

(A.6) Φaaab=∑A(32(∑Type  1(12)Ltriple−1ΦAAA+∑Type  2(12)LtripleΦAA)+∑Type  3(12)Lpair+2ΦAA),

where *A*: a common ancestor of *a* and *b*.

When there is only one mergeable path-pair (let us consider 〈*P*_*Aa*1_, *P*_*Aa*2_〉 as the mergeable path-pair),

Type 1: 〈*P*_*Aa*1_, *P*_*Aa*3_, *P*_*Ab*_〉 has zero root 2-overlap path,
Type 2: 〈*P*_*Aa*1_, *P*_*Aa*3_, *P*_*Ab*_〉 has one root 2-overlap path *P*_*As*_ ending at *s*.

When 〈*P*_*Aa*1_, *P*_*Aa*2_, *P*_*Aa*3_〉 is mergeable,

Type 3: 〈*P*_*Aa*_, *P*_*Ab*_〉 is nonoverlapping
  (A.7) Ltriple={LPAa1+LPAa3+LPAbfor  Type  1LPAa1+LPAa3+LPAb−LPAsfor  Type  2,Lpair=LPAa+LPAb for    Type  3.

Note that if *a* is an ancestor of *b*, we treat Φ_*aa**ab*_ = Φ_*a*1*a*2*a*3*b*_. Then, we apply the path-counting formula for Φ_*ab**cd*_ to compute Φ_*a*1*a*2*a*3*b*_.

### B. Proof for Path-Counting Formulas of Three Individuals

We first demonstrate that, for one triple-common ancestor *A*, the path-counting computation of Φ_*ab**c*_ is equivalent to the computation using recursive formulas. Then, we prove the correctness of the path-counting computation for multiple triple-common ancestors.

#### B.1. One Triple-Common Ancestor

Considering the different types of path-triples starting from a triple-common ancestor *A* in a pedigree graph *G* contributing to Φ_*ab**c*_ and Φ_*a**ab*_, *G* can have 5 different cases:

(B.1) Case  2.1:  G  does not have   any  path-triples 〈PAa1,PAa2,PAb〉  with  root  overlapCase  2.2:  G  has path-triples  〈PAa1,PAa2,PAb〉with  root  overlapCase  2.3:  G  has path-triples  〈PAa1,PAa2,PAb〉 having  mergeable  path-pair〈PAa1,PAa2〉}⟸Φaab,Case  3.1:  G  does not have any path-triples  〈PAa,PAb,PAc〉  with  root  overlapCase  3.2:  G  has path-triples  〈PAa,PAb,PAc〉  with  root  overlap}⟸Φabc.

Based on the 5 cases from Case 2.1 to Case 3.2, we first construct a dependency graph shown in Figure 17, consistent with the recursive formulas (3), (4), and (5) for the generalized kinship coefficients for three individuals.

Then, we take the following steps to prove the correctness of the path-counting formulas (12) and (A.1):

- for Φ_*ab*_, the correctness of the path-counting formula (i.e., Wright's formula) is proven in [21]. For Case 2.1 and Case 2.2, the correctness is proven based on the correctness of Cases 3.1 and 3.2;
- for Case 2.3, it has no cycle but only depends on Φ_*ab*_. Thus, we prove the correctness of Case 2.3 by transforming the case to Φ_*ab*_;
- for Cases 3.1 and 3.2, the correctness is proven by induction on the number of edges, *n*, in the pedigree graph *G*.

##### B.1.1. Correctness Proof for Case 3.1

*Case 3.1.* For Φ_*ab**c*_, *G* does not have any path triples 〈*P*_*Aa*_, *P*_*Ab*_, *P*_*Ac*_〉 with root overlap.

Proof There are two basic scenarios: (i) one individual is a parent of another; (ii) no individual is a parent of another, among *a*, *b*, and *c*. Using the recursive formula (3) to compute Φ_*ab**c*_, for Figure 18(a), Φ_*ab**c*_ = (1/2)Φ_*c**bc*_ = (1/2)^2^Φ_*ccc*_; for Figure 18(b), Φ_*ab**c*_ = (1/2)Φ_*Ab**c*_ = (1/2)^2^Φ_*A**Ac*_ = (1/2)^3^Φ_*AA**A*_. Using the path-counting formula (12), if a path-triple 〈*P*_*Aa*_, *P*_*Ab*_, *P*_*Ac*_〉 has no root overlap (i.e., Type 1), then the contribution of 〈*P*_*Aa*_, *P*_*Ab*_, *P*_*Ac*_〉 to Φ_*ab**c*_ can be computed as follows: ∑_Type  1_(1/2)^*L*_〈*P*_*Aa*_,*P*_*Ab*_,*P*_*Ac*_〉_^Φ_*AA**A*_, where *L*_〈*P*_*Aa*_,*P*_*Ab*_,*P*_*Ac*_〉_ = *L*_*P*_*Aa*__ + *L*_*P*_*Ab*__ + *L*_*P*_*Ac*__. For Figure 18(a), *c* is the only triple-common ancestor and we obtain Φ_*ab**c*_ = (1/2)^*L*_〈*P*_*ca*_,*P*_*cb*_,*P*_*cc*_〉_^Φ_*ccc*_ = (1/2)^2^Φ_*ccc*_; for Figure 18(b), we obtain Φ_*ab**c*_ = (1/2)^*L*_〈*P*_*Aa*_,*P*_*Ab*_,*P*_*Ac*_〉_^Φ_*AA**A*_ = (1/2)^3^Φ_*AA**A*_.
*Induction Step.* Let *n* denote the number of edges in *G*. Assume true for *n* ≤ *k*, where *k* ≥ 2. Then, we show it is true for *n* = *k* + 1. For Figures 19(a) and 19(b), among *a*, *b*, and *c*, let *a* be the individual having the longest path starting from their triple-common ancestor in the pedigree graph *G* with (*k* + 1) edges. If we remove the node *a* and cut the edge *f* → *a* from *G*, then the new graph *G** has *k* edges. In terms of computing Φ_*f**bc*_, *G** satisfies the condition for induction hypothesis. For Figure 19(a), Φ_*f**bc*_ = ∑_Type  1_(1/2)^*L*_〈*P*_*Af*_,*P*_*Ab*_,*P*_*Ac*_〉_^Φ_*AA**A*_. Based on the recursive formula (3), Φ_*ab**c*_ = (1/2)(Φ_*f**bc*_ + Φ_*m**bc*_) where *f* and *m* are parents of *a*. In *G*, *a* only has one parent *f*; thus, it indicates Φ_*m**bc*_ = 0. Then, we can plug-in the path-counting formula for Φ_*f**bc*_ to obtain

(B.2) Φabc=12Φfbc=12∗∑Type  1(12)L〈PAf,PAb,PAc〉ΦAAA=∑Type  1(12)L〈PAf,PAb,PAc〉+1ΦAAA∵L〈PAa,PAb,PAc〉=L〈PAf,PAb,PAc〉+1∴Φabc=∑Type  1(12)L〈PAa,PAb,PAc〉ΦAAA.

Similarly, for Figure 19(b), we obtain Φ_*ab**c*_ = ∑_Type  1_(1/2)^*L*_〈*P*_*cf*_,*P*_*cb*_,*P*_*cc*_〉_+1^Φ_*ccc*_ = ∑_Type  1_(1/2)^*L*_〈*P*_*ca*_,*P*_*cb*_,*P*_*cc*_〉_^Φ_*ccc*_. Thus, it is true for *n* = *k* + 1.

##### B.1.2. Correctness Proof for Case 3.2

*Case 3.2.* For Φ_*ab**c*_, *G* has path triples 〈*P*_*Aa*_, *P*_*Ab*_, *P*_*Ac*_〉 with root overlap.

Proof There are three basic scenarios: (i) there are two individuals who are parents of another; (ii) there is only one individual who is parent of another; (iii) there is no individual who is a parent of another, among *a*, *b*, and *c*. Using the recursive formula (3) to compute Φ_*ab**c*_: in Figure 20, for Figure 20(a), Φ_*ab**c*_ = (1/2)Φ_*b**bc*_ = (1/2)^2^Φ_*bc*_ = (1/2)^3^Φ_*cc*_; for Figure 20(b),Φ_*ab**c*_ = (1/2)Φ_*b**bc*_ = (1/2)^2^Φ_*bc*_ = (1/2)^4^Φ_*AA*_; for Figure 20(c), Φ_*ab**c*_ = (1/2)^2^Φ_*ssc*_ = (1/2)^3^Φ_*sc*_ = (1/2)^5^Φ_*AA*_. Using the path-counting formula (12), if a path-triple 〈*P*_*Aa*_, *P*_*Ab*_, *P*_*Ac*_〉 has root overlap (i.e., Type 2), then the contribution of 〈*P*_*Aa*_, *P*_*Ab*_, *P*_*Ac*_〉 to Φ_*ab**c*_ can be computed as follows:∑_Type  2_(1/2)^*L*_〈*P*_*Aa*_,*P*_*Ab*_,*P*_*Ac*_〉_+1^Φ_*AA*_, where *L*_〈*P*_*Aa*_,*P*_*Ab*_,*P*_*Ac*_〉_ = *L*_*P*_*Aa*__ + *L*_*P*_*Ab*__ + *L*_*P*_*Ac*__ − *L*_*P*_*As*__and *s* is the last individual of the root overlap path *P*_*As*_. For Figure 20(a), *c* is the only triple-common ancestor and we obtain Φ_*ab**c*_ = (1/2)^*L*_〈*P*_*ca*_,*P*_*cb*_,*P*_*cc*_〉_+1^Φ_*cc*_ = (1/2)^2+1^Φ_*cc*_ = (1/2)^3^Φ_*cc*_. Similarly, for Figures 20(b) and 20(c), we obtain Φ_*ab**c*_ = (1/2)^4^Φ_*AA*_ and Φ_*ab**c*_ = (1/2)^5^Φ_*AA*_, respectively.
*Induction Step*. Let *n* denote the number of edges in *G*. Assume true for *n* ≤ *k*, where *k* ≥ 2. Show that it is true for = *k* + 1. For Figures 21(a), 21(b), and 21(c), among *a*, *b*, and *c*, let *a* be the individual who has the longest path and let *p* be a parent of *a*. Then, we cut the edge *p* → *a* from *G* and obtain a new graph *G** which satisfies the condition of induction hypothesis. For Figure 21(a), we use the path-counting formula for Φ_*f**bc*_ in *G** : Φ_*f**bc*_ = ∑_Type  2_(1/2)^*L*_〈*P*_*Af*_,*P*_*Ab*_,*P*_*Ac*_〉_+1^Φ_*AA*_. In *G*, *f* is the only parent of *a*, according to the recursive formula (3), we have Φ_*ab**c*_ = (1/2)Φ_*f**bc*_. Then, we can plug-in the Φ_*f**bc*_ and obtain

(B.3) Φabc=12Φfbc=12∑Type  2(12)L〈PAf,PAb,PAc〉+1ΦAA=∑Type  2(12)L〈PAf,PAb,PAc〉+1+1ΦAA∵L〈PAa,PAb,PAc〉=L〈PAf,PAb,PAc〉+1∴Φabc=∑Type  2(12)L〈PAf,PAb,PAc〉+1+1ΦAA=∑Type  2(12)L〈PAa,PAb,PAc〉+1ΦAA.

For Figures 21(b) and 21(c), we take the same steps as we calculate Φ_*ab**c*_ for Figure 21(a). In summary, it is true for *n* = *k* + 1.

##### B.1.3. Correctness Proof for Case 2.3

*Case 2.3.* For Φ_*a**ab*_, the path-triples in the pedigree graph *G* have mergeable path-pair.

Proof Considering the relationship between *a* and *b*, *G* has two scenarios: (i) *b* is not an ancestor of *a*; (ii) *b* is an ancestor of *a*. Using the path-counting formula (A.1), if a path-triple 〈*P*_*Aa*1_, *P*_*Aa*2_, *P*_*Ab*_〉∈ Type 3, which means that it has a mergeable path-pair, then the contribution of 〈*P*_*Aa*1_, *P*_*Aa*2_, *P*_*Ab*_〉 to Φ_*a**ab*_ can be computed as follows: ∑_Type  3_(1/2)^*L*_〈*P*_*Aa*_,*P*_*Ab*_〉_+1^Φ_*AA*_, where *L*_〈*P*_*Aa*_,*P*_*Ab*_〉_ = *L*_*P*_*Aa*__ + *L*_*P*_*Ab*__. Using the recursive formula (4), we obtain Φ_*a**ab*_ = (1/2)(Φ_*ab*_ + Φ_*fmb*_). For Figure 22(a), *A* is a common ancestor of *a* and *b*. ∵*a*  only  has  one  parent  *f*

(B.4) ∴Φaab=12(Φab+Φfmb)=12(Φab+0)=12Φab (as  m  is  missing).

For Φ_*ab*_, we use Wright's formula and obtain Φ_*ab*_ = ∑_*P*_(1/2)^*L*_〈*P*_*Aa*_,*P*_*Ab*_〉_^Φ_*AA*_ where *P* denotes all nonoverlapping path-pairs 〈*P*_*Aa*_, *P*_*Ab*_〉. Then, we have Φ_*a**ab*_ = (1/2)Φ_*ab*_ = (1/2)∑_*P*_(1/2)^*L*_〈*P*_*Aa*_,*P*_*Ab*_〉_^Φ_*AA*_ = ∑_*P*_(1/2)^*L*_〈*P*_*Aa*_,*P*_*Ab*_〉_+1^Φ_*AA*_. For Figure 22(b), we can also transform the computation of Φ_*a**ab*_ to Φ_*ab*_. In summary, it shows that the path-counting formula (A.1) is true for Case 2.3.

##### B.1.4. Correctness Proof for Cases 2.1 and 2.2

For Φ_*a**ab*_, when there is no path-triple having mergeable path-pair, (i.e., the path-triple belongs to either Case 2.1 or Case 2.3), Φ_*a**ab*_ can be transformed to Φ_*a*_1_*a*_2_*b*_, which is equivalent to the computation of Φ_*ab**c*_ for Cases 3.1 and 3.2. The correctness of our path-counting formula for Cases 3.1 and 3.2 is proven. Thus, we obtain the correctness for Φ_*a**ab*_ when the path-triple belongs to either Case 2.1 or Case 2.2.

#### B.2. Multiple Triple-Common Ancestors

Now, we provide the correctness proof for multiple triple-common ancestors regarding the path-counting formulas (12) and (A.1).

Lemma B.2 A. Given a pedigree graph *G* and three individuals *a*, *b*, *c* having at least one trip-common ancestor, Φ_*ab**c*_ is correctly computed using the path counting formulas (12) and (A.1).

Proof
Proof by induction on the number of triple-common ancestors
*Basis. G* has only one triple-common ancestor of *a*, *b*, and *c*. The correctness of (12) and (A.1) for *G* with only one triple-common ancestor of *a*, *b*, and *c* is proven in the previous section.
*Induction Hypothesis.* Assume that if *G* has *k* or less triple-common ancestors of *a*, *b*, and *c*, (12) and (A.1) are correct for *G*. 
*Induction Step.* Now, we show that it is true for *G* with *k* + 1 triple-common ancestors of *a*, *b*, and *c*. Let *Tri*_*C*(*a*, *b*, *c*, *G*) denote all triple-common ancestors of *a*, *b*, and *c* in *G*, where *Tri*_*C*(*a*, *b*, *c*, *G*) = {*A*_*i*_∣1 ≤ *i* ≤ *k* + 1}. Let *A*_1_ be the most top triple-common ancestor such that there is no individual among the remaining ancestors {*A*_*i*_∣2 ≤ *i* ≤ *k* + 1} who is an ancestor of *A*_1_.   Let *S*(*A*_1_) denote the contribution from *A*_1_ to Φ_*ab**c*_. Because *A*_1_ is the most top triple-common ancestor, there is no path-triple from {*A*_*i*_∣2 ≤ *i* ≤ *k* + 1} to *a*, *b*, and *c* which passes through *A*_1_. Then, we can remove *A*_1_ from *G* and delete all out-going edges from *A*_1_ and obtain a new graph *G*′ which has *k* triple-common ancestors of *a*, *b*, and *c*. It means *Tri*_*C*(*a*, *b*, *c*, *G*′) = {*A*_*i*_∣2 ≤ *i* ≤ *k* + 1}. For the new graph *G*′, we can apply our induction hypothesis and obtain Φ_*ab**c*_^  ^  (*G*′). For the most top triple-common ancestor *A*_1_, there are two different cases considering its relationship with the other triple-common ancestors:

1. there is no individual among {*A*_*i*_∣2 ≤ *i* ≤ *k* + 1} who is a descendant of *A*_1_;
2. there is at least one individual among {*A*_*i*_∣2 ≤ *i* ≤ *k* + 1} who is a descendant of *A*_1_.

For (1), since no individual among {*A*_*i*_∣2 ≤ *i* ≤ *k* + 1} is a descendant of *A*_1_, the set of path-triples from *A*_1_ to *a*, *b*, and *c* is independent of the set of path-triples from {*A*_*i*_∣2 ≤ *i* ≤ *k* + 1} to *a*, *b*, and *c*. It also means that the contribution from *A*_1_ to Φ_*ab**c*_^  ^  is independent of the contribution from the other triple-common ancestors. Summing up all contributions, we can obtain Φ_*ab**c*_^  ^  (*G*) = Φ_*ab**c*_^  ^  (*G*′) + *S*(*A*_1_). For (2), let *A*_*j*_ be one descendant of *A*_1_. Now both *A*_1_ and *A*_*j*_ can reach *a*, *b*, and *c*.
*pt*_*i*_ = {*t*_*a*_:  *A*_1_ → ⋯→*a*; *t*_*b*_:  *A*_1_ → ⋯→*b*; *t*_*c*_:  *A*_1_ → ⋯→*c*}, a path-triple from *A*_1_ to *a*, *b*, and *c*. If *t*_*a*_, *t*_*b*_, and *t*_*c*_ all pass through *A*_*j*_, then the path-triple *pt*_*i*_ is not an eligible path-triple for Φ_*ab**c*_. When we compute the contribution from *A*_1_ to Φ_*ab**c*_, we exclude all such path-triples where *t*_*a*_, *t*_*b*_, and *t*_*c*_ all pass through a lower triple-common ancestor. In other words, an eligible path-triple from *A*_1_ regarding Φ_*ab**c*_ cannot have three paths all passing through a lower triple-common ancestor. Therefore, we know that that the contribution from *A*_1_ to Φ_*ab**c*_ is independent of the contribution from the other triple-common ancestors. Summing up all contributions, we obtain Φ_*ab**c*_(*G*) = Φ_*ab**c*_(*G*′) + *S*(*A*_1_).

### C. Proof for Four Individuals and Two Pairs of Individuals

Here, we give a proof sketch for the correctness of path counting formulas for four individuals. First of all, for four individuals in a pedigree graph *G*, we present all different cases based on which we construct a dependency graph. The correctness of the path-counting formulas for two-pair individuals can be proved similarly.

#### C.1. Proof for Four Individuals

Consider the existence of different types of path-quads regarding Φ_*ab**cd*_, Φ_*a**ab**c*_, and Φ_*aa**ab*_; there are 15 cases for a pedigree graph *G*:

(C.1) Case  2.1:  G  has  path-triples 〈PAa1,PAa2,PAb〉 with  zero  root  overlapCase  2.2:  G  has  path-triples 〈PAa1,PAa2,PAb〉 with  one  root  overlapCase  2.3:  G  has  path-pairs  〈PAa,PAb〉 with  zero  root  overlap}⟸Φaaab,Case  3.1:  G  has  path-quads 〈PAa1,PAa2,PAb,PAc〉 with  zero  root  overlapCase  3.2:  G  has  path-quads 〈PAa1,PAa2,PAb,PAc〉 with  one  root  2-overlapCase  3.3.1:  G  has  path-quads 〈PAa1,PAa2,PAb,PAc〉 with  two  root  2-overlapCase  3.3.2:  G  has  path-quads 〈PAa1,PAa2,PAb,PAc〉 with  one  root  3-overlapCase  3.3.3:  G  has  path-quads 〈PAa1,PAa2,PAb,PAc〉 with  one  root 2-overlap   and  one root 3-overlapCase  3.4:  G  has  path-triples 〈PAa,PAb,PAc〉 with  zero  root  overlapCase  3.5:  G  has  path-triples 〈PAa,PAb,PAc〉 with  one  root  overlap}⟸Φaabc,Case  4.1:  G  has  path-quads 〈PAa,PAb,PAc,PAd〉 with  zero  root  overlapCase  4.2:  G  has  path-quads 〈PAa,PAb,PAc,PAd〉 with  one  root  2-overlapCase  4.3.1:  G  has  path-quads 〈PAa,PAb,PAc,PAd〉 with  two  root  2-overlapCase  4.3.2:  G  has  path-quads 〈PAa,PAb,PAc,PAd〉 with  one  root  3-overlapCase  4.3.3:  G  has  path-quads 〈PAa,PAb,PAc,PAd〉 with one root 2-overlap  and one root 3-overlap}⟸Φabcd.

Then, we construct a dependency graph shown in Figure 23 for all cases for four individuals.

According to the dependency graph in Figure 23, the intermediate steps including Cases 3.4 and 3.5 are already proved for the computation of Φ_*ab**c*_. The correctness of the transformation from Case 4.2 to Case 3.4 can be proved based on the recursive formula for Φ_*ab**cd*_ and Φ_*a**ab**c*_. Similarly, we can obtain the transformation from Case 4.3.1 to Case 3.5.

#### C.2. Proof for Two Pairs of Individuals

Consider the existence of different types of 2-pair-path-pair regarding Φ_*ab*,*cd*_; there are 9 cases which are listed as follows.

*Case 4.1. G*  has 〈(*P*_*Aa*_, *P*_*Ab*_), (*P*_*Ac*_, *P*_*Ad*_)〉 with zero root homo-overlap and zero root heter-overlap.

*Case 4.2*. *G*  has 〈(*P*_*Aa*_, *P*_*Ab*_), (*P*_*Ac*_, *P*_*Ad*_)〉 with zero root homo-overlap and one root heter-overlap.

*Case 4.3.1. G*  has 〈(*P*_*Aa*_, *P*_*Ab*_), (*P*_*Ac*_, *P*_*Ad*_)〉 with zero root homo-overlap and two root heter-overlap.

*Case 4.3.2.G*  has 〈(*P*_*Aa*_, *P*_*Ab*_), (*P*_*Ac*_, *P*_*Ad*_)〉 with one root homo-overlap and two root heter-overlap.

*Case 4.4.* *G*  has 〈(*P*_*Aa*_, *P*_*Ab*_), (*P*_*Ac*_, *P*_*Ad*_)〉 with one root homo-overlap and zero root heter-overlap.

*Case 4.5. G*  has 〈(*P*_*Aa*_, *P*_*Ab*_), (*P*_*Ac*_, *P*_*Ad*_)〉 with two root homo-overlap and zero root heter-overlap.

*Case 4.6. G*  has path-triples 〈*P*_*Aa*_, *P*_*Ab*_, *P*_*Ac*_〉 with zero root overlap.

*Case 4.7. G*  has path-triples 〈*P*_*Aa*_, *P*_*Ab*_, *P*_*Ac*_〉 with one root overlap.

*Case 4.8. G*  has path-pairs 〈*P*_*Tc*_, *P*_*Td*_〉 with zero root overlap.

Then, we construct a dependency graph for the cases relating to Φ_*ab*,*cd*_ in Figure 24.

According to the dependency graph in Figure 24, Cases 4.6,  4.7, and 4.8 are the intermediate steps which already are proved for the computation of Φ_*ab**c*_. The correctness of the transformation from Case 4.2 to Case 4.6 can be proved based on the recursive formula for Φ_*ab*,*cd*_ and Φ_*ab*,*ac*_. Similarly, we can obtain the transformation from Cases 4.3.1 and 4.3.2 to Case 4.7 as well as from Case 4.4 to Case 4.8 accordingly.

## References

[1] http://compmed.com/category/people-helping-people/page/7/.

[2] Falchi M, Forabosco P, Mocci E (2004). A genomewide search using an original pairwise sampling approach for large genealogies identifies a new locus for total and low-density lipoprotein cholesterol in two genetically differentiated isolates of Sardinia. *The American Journal of Human Genetics*.

[3] Ciullo M, Bellenguez C, Colonna V (2006). New susceptibility locus for hypertension on chromosome 8q by efficient pedigree-breaking in an Italian isolate. *Human Molecular Genetics*.

[4] http://www.genome.gov/glossary/?id=148.

[5] Cotterman CW (1940). *A calculus for statistico-genetics [Ph.D. thesis]*.

[6] Malecot G (1948). *Les mathématique de l'hérédité*.

[7] Gillois M (1964). La relation d'identité en génétique. *Annales de l'Institut Henri Poincaré B*.

[8] Harris DL (1964). Genotypic covariances between inbred relatives. *Genetics*.

[9] Jacquard A (1966). Logique du calcul des coefficients d’identite entre deux individuals. *Population*.

[10] Karigl G (1981). A recursive algorithm for the calculation of identity coefficients. *Annals of Human Genetics*.

[11] Elliott B, Akgul SF, Mayes S, Ozsoyoglu ZM Efficient evaluation of inbreeding queries on pedigree data.

[12] Elliott B, Cheng E, Mayes S, Ozsoyoglu ZM (2009). Efficiently calculating inbreeding on large pedigrees databases. *Information Systems*.

[13] Yang L, Cheng E, Özsoyoğlu ZM Using compact encodings for path-based computations on pedigree graphs.

[14] Cheng E, Elliott B, Ozsoyoglu ZM Scalable computation of kinship and identity coefficients on large pedigrees.

[15] Cheng E, Elliott B, Özsoyoĝlu ZM (2009). Efficient computation of kinship and identity coefficients on large pedigrees. *Journal of Bioinformatics and Computational Biology (JBCB)*.

[16] Wright S (1922). Coefficients of inbreeding and relationship. *The American Naturalist*.

[17] Nadot R, Vaysseix G (1973). Kinship and identity algorithm of coefficients of identity. *Biometrics*.

[18] Cheng E (2012). *Scalable path-based computations on pedigree data [Ph.D. thesis]*.

[19] Ollikainen V (2002). *Simulation Techniques for Disease Gene Localization in Isolated Populations [Ph.D. thesis]*.

[20] Toivonen HTT, Onkamo P, Vasko K (2000). Data mining applied to linkage diseqilibrium mapping. *The American Journal of Human Genetics*.

[21] Boucher W (1988). Calculation of the inbreeding coefficient. *Journal of Mathematical Biology*.

